# FRET-based dynamic structural biology: Challenges, perspectives and an appeal for open-science practices

**DOI:** 10.7554/eLife.60416

**Published:** 2021-03-29

**Authors:** Eitan Lerner, Anders Barth, Jelle Hendrix, Benjamin Ambrose, Victoria Birkedal, Scott C Blanchard, Richard Börner, Hoi Sung Chung, Thorben Cordes, Timothy D Craggs, Ashok A Deniz, Jiajie Diao, Jingyi Fei, Ruben L Gonzalez, Irina V Gopich, Taekjip Ha, Christian A Hanke, Gilad Haran, Nikos S Hatzakis, Sungchul Hohng, Seok-Cheol Hong, Thorsten Hugel, Antonino Ingargiola, Chirlmin Joo, Achillefs N Kapanidis, Harold D Kim, Ted Laurence, Nam Ki Lee, Tae-Hee Lee, Edward A Lemke, Emmanuel Margeat, Jens Michaelis, Xavier Michalet, Sua Myong, Daniel Nettels, Thomas-Otavio Peulen, Evelyn Ploetz, Yair Razvag, Nicole C Robb, Benjamin Schuler, Hamid Soleimaninejad, Chun Tang, Reza Vafabakhsh, Don C Lamb, Claus AM Seidel, Shimon Weiss

**Affiliations:** 1 Department of Biological Chemistry, The Alexander Silberman Institute of Life Sciences, and The Center for Nanoscience and Nanotechnology, Faculty of Mathematics & Science, The Edmond J. Safra Campus, The Hebrew University of JerusalemJerusalemIsrael; 2 Lehrstuhl für Molekulare Physikalische Chemie, Heinrich-Heine-UniversitätDüsseldorfGermany; 3 Dynamic Bioimaging Lab, Advanced Optical Microscopy Centre and Biomedical Research Institute (BIOMED), Hasselt UniversityDiepenbeekBelgium; 4 Department of Chemistry, University of SheffieldSheffieldUnited Kingdom; 5 Department of Chemistry and iNANO center, Aarhus UniversityAarhusDenmark; 6 Department of Structural Biology, St. Jude Children's Research HospitalMemphisUnited States; 7 Laserinstitut HS Mittweida, University of Applied Science MittweidaMittweidaGermany; 8 Laboratory of Chemical Physics, National Institute of Diabetes and Digestive and Kidney Diseases, National Institutes of HealthBethesdaUnited States; 9 Physical and Synthetic Biology, Faculty of Biology, Ludwig-Maximilians-Universität MünchenPlanegg-MartinsriedGermany; 10 Department of Integrative Structural and Computational Biology, The Scripps Research InstituteLa JollaUnited States; 11 Department of Cancer Biology, University of Cincinnati School of MedicineCincinnatiUnited States; 12 Department of Biochemistry and Molecular Biology and The Institute for Biophysical Dynamics, University of ChicagoChicagoUnited States; 13 Department of Chemistry, Columbia UniversityNew YorkUnited States; 14 Department of Biophysics and Biophysical Chemistry, Department of Biomedical Engineering, Johns Hopkins University School of Medicine, Howard Hughes Medical InstituteBaltimoreUnited States; 15 Department of Chemical and Biological Physics, Weizmann Institute of ScienceRehovotIsrael; 16 Department of Chemistry & Nanoscience Centre, University of CopenhagenCopenhagenDenmark; 17 Denmark Novo Nordisk Foundation Centre for Protein Research, Faculty of Health and Medical Sciences, University of CopenhagenCopenhagenDenmark; 18 Department of Physics and Astronomy, and Institute of Applied Physics, Seoul National UniversitySeoulRepublic of Korea; 19 Center for Molecular Spectroscopy and Dynamics, Institute for Basic Science and Department of Physics, Korea UniversitySeoulRepublic of Korea; 20 Institute of Physical Chemistry and Signalling Research Centres BIOSS and CIBSS, University of FreiburgFreiburgGermany; 21 Department of Chemistry and Biochemistry, and Department of Physiology, University of California, Los AngelesLos AngelesUnited States; 22 Department of BioNanoScience, Kavli Institute of Nanoscience, Delft University of TechnologyDelftNetherlands; 23 Biological Physics Research Group, Clarendon Laboratory, Department of Physics, University of OxfordOxfordUnited Kingdom; 24 School of Physics, Georgia Institute of TechnologyAtlantaUnited States; 25 Physical and Life Sciences Directorate, Lawrence Livermore National LaboratoryLivermoreUnited States; 26 School of Chemistry, Seoul National UniversitySeoulRepublic of Korea; 27 Department of Chemistry, Pennsylvania State UniversityUniversity ParkUnited States; 28 Departments of Biology and Chemistry, Johannes Gutenberg UniversityMainzGermany; 29 Institute of Molecular Biology (IMB)MainzGermany; 30 Centre de Biologie Structurale (CBS), CNRS, INSERM, Universitié de MontpellierMontpellierFrance; 31 Institüt of Biophysics, Ulm UniversityUlmGermany; 32 Department of Biophysics, Johns Hopkins UniversityBaltimoreUnited States; 33 Department of Biochemistry and Department of Physics, University of ZurichZurichSwitzerland; 34 Department of Bioengineering and Therapeutic Sciences, University of California, San FranciscoSan FranciscoUnited States; 35 Physical Chemistry, Department of Chemistry, Center for Nanoscience (CeNS), Center for Integrated Protein Science Munich (CIPSM) and Nanosystems Initiative Munich (NIM), Ludwig-Maximilians-UniversitätMünchenGermany; 36 Warwick Medical School, University of WarwickCoventryUnited Kingdom; 37 Biological Optical Microscopy Platform (BOMP), University of MelbourneParkvilleAustralia; 38 College of Chemistry and Molecular Engineering, PKU-Tsinghua Center for Life Sciences, Beijing National Laboratory for Molecular Sciences, Peking UniversityBeijingChina; 39 Department of Molecular Biosciences, Northwestern UniversityEvanstonUnited States; 40 Department of Physiology, CaliforniaNanoSystems Institute, University of California, Los AngelesLos AngelesUnited States; Weill Cornell MedicineUnited States; Weill Cornell MedicineUnited States

**Keywords:** FRET, single-molecule, conformation, dynamics, biomolecules, community

## Abstract

Single-molecule FRET (smFRET) has become a mainstream technique for studying biomolecular structural dynamics. The rapid and wide adoption of smFRET experiments by an ever-increasing number of groups has generated significant progress in sample preparation, measurement procedures, data analysis, algorithms and documentation. Several labs that employ smFRET approaches have joined forces to inform the smFRET community about streamlining how to perform experiments and analyze results for obtaining quantitative information on biomolecular structure and dynamics. The recent efforts include blind tests to assess the accuracy and the precision of smFRET experiments among different labs using various procedures. These multi-lab studies have led to the development of smFRET procedures and documentation, which are important when submitting entries into the archiving system for integrative structure models, PDB-Dev. This position paper describes the current ‘state of the art’ from different perspectives, points to unresolved methodological issues for quantitative structural studies, provides a set of ‘soft recommendations’ about which an emerging consensus exists, and lists openly available resources for newcomers and seasoned practitioners. To make further progress, we strongly encourage ‘open science’ practices.

## Introduction

Understanding how biomolecules couple structural dynamics with function is at the heart of several disciplines and remains an outstanding goal in biology. Linking conformational states and their transitions to biochemical function requires the ability to precisely resolve the structure and dynamics of a biological system, which is often altered upon ligand binding or influenced by the chemical and physical properties of its environment. The most well-established structural biology tools have provided high-resolution ‘snapshots’ of states in a crystallized or frozen form (e.g., X-ray crystallography and single-particle cryo-electron microscopy, cryoEM) or an ensemble average of all contributing conformations (e.g., nuclear magnetic resonance, NMR; small-angle X-ray scattering, SAXS; small-angle neutron scattering, SANS; double electron-electron resonance, DEER; cross-linking mass spectrometry, XL-MS; ensemble-FRET). In recent years, further developments have enabled these conventional structural tools to detect conformational dynamics and reaction intermediates. For example, NMR techniques (Anthis and Clore, 2015; Clore and Iwahara, 2009; Palmer, 2004; Ravera et al., 2014; Sekhar and Kay, 2019) and electron paramagnetic resonance techniques (Jeschke, 2018; Jeschke, 2012; Krstić et al., 2011) have been advanced to study conformational dynamics and capture transient intermediates. Time-resolved crystallographic investigations have been employed to resolve functionally relevant structural displacements associated with a biological function (Kupitz et al., 2014; Moffat, 2001; Schlichting et al., 1990; Schlichting and Chu, 2000; Schotte et al., 2003). Advances in microfluidic mixing and spraying devices have enabled time-resolved cryoEM (Feng et al., 2017; Kaledhonkar et al., 2018) and cross-linking mass spectrometry (XL-MS or CL-MS) (Braitbard et al., 2019; Brodie et al., 2019; Chen et al., 2020; Iacobucci et al., 2019; Murakami et al., 2013; Slavin and Kalisman, 2018). Progress in computational methods has also afforded novel tools for examining biomolecular structure and dynamics. Each of these advances highlights an increased awareness that one needs to directly and continuously track the dynamical properties of individual biomolecules in order to understand their function and regulation.

In this context, FRET (referred to as fluorescence resonance energy transfer or Förster resonance energy transfer [Braslavsky et al., 2008]) studies at the ensemble and single-molecule levels have emerged as important tools for measuring structural dynamics over at least 12 orders of magnitude in time and mapping the conformational and functional heterogeneities of biomolecules under ambient conditions. FRET studies probing fluorescence decays at the ensemble level (Grinvald et al., 1972; Haas et al., 1975; Haas and Steinberg, 1984; Hochstrasser et al., 1992) (time-resolved FRET) permitted already in the early 1970s the study of structural heterogeneities on timescales longer than the fluorescence lifetime (a few ns). This approach is still used nowadays (Becker, 2019; Orevi et al., 2014; Peulen et al., 2017) and has been transferred to single-molecule studies. The ability to measure FRET in single molecules (Deniz et al., 1999; Ha et al., 1996; Lerner et al., 2018a) has made the method even more appealing. The single-molecule FRET (smFRET) approach has been extensively used to study conformational dynamics and biomolecular interactions under steady-state conditions (Dupuis et al., 2014; Larsen et al., 2019; Lerner et al., 2018a; Lipman et al., 2003; Margittai et al., 2003; Mazal and Haran, 2019; Michalet et al., 2006; Orevi et al., 2014; Ray et al., 2019; Sasmal et al., 2016; Schuler et al., 2005; Schuler et al., 2002; Steiner et al., 2008; Zhuang et al., 2000). It is notable that, in many mechanistic studies, it suffices to use FRET for distinguishing different conformations and determining kinetic rates such that absolute FRET efficiencies and thereby distances do not need to be determined. However, the ability to measure accurate distances and kinetics with smFRET has led to its emergence as an important tool in this new era of ‘*dynamic structural biology*’ for mapping biomolecular heterogeneities and for measuring structural dynamics over a wide range of timescales (Lerner et al., 2018a; Mazal and Haran, 2019; Sanabria et al., 2020; Schuler and Hofmann, 2013; Weiss, 1999).

**Figure 1.**
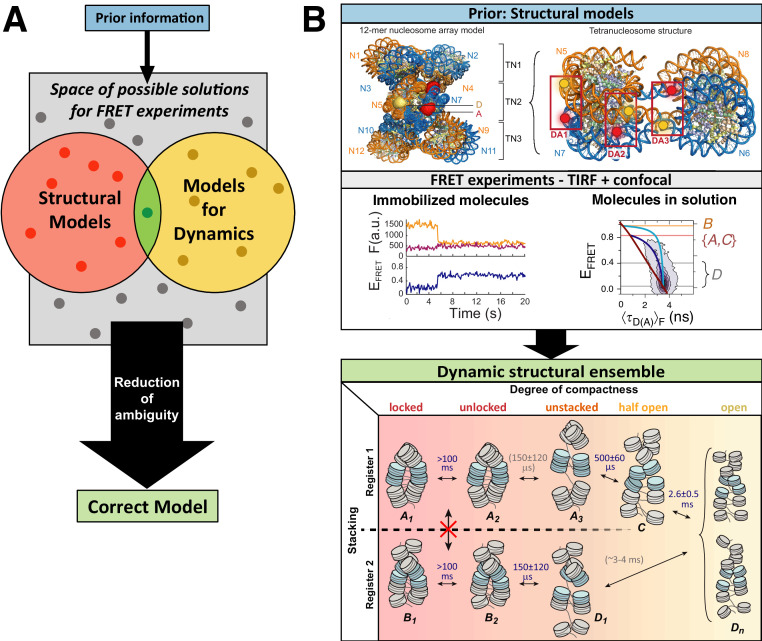
Workflow of modeling dynamic structures from FRET measurements. (**A**) Integrative modeling requires structural and dynamic information. Prior information from conventional approaches (X-ray, NMR, cryoEM) together with computational tools defines the space of possible solutions for FRET-assisted structural modeling. The combination of structural (inter-dye distances) and dynamic information (kinetic connectivity and exchange rates) enables identification of a consistent model. (**B**) Study of structure and dynamics of chromatin fibers. A combined TIRF and confocal FRET study of structure and dynamics of chromatin fibers using three FRET labeling positions (DA1-3) for two pairs of dyes with distinct Förster distances. Förster distances ( is defined in section Inter-dye distances, Equation 6). Prior structural information provided by cryo-electron microscopy (top, left) (Song et al., 2014) and X-ray crystallography (top, right PDB ID: 1ZBB Schalch et al., 2005) is combined with the structural and dynamic information obtained by FRET experiments on immobilized molecules measured by total internal reflection (TIRF) microscopy and on freely diffusing molecules by confocal microscopy (Kilic et al., 2018). From the combined information, a consistent model is derived for chromatin fiber conformations with shifted registers, which are connected by slow (>100 ms) and fast de-compaction processes (150 µs) that do not proceed directly, but rather through an open fiber conformation. Figure 1B was reproduced from Figures 1, 3, and 6 in Kilic et al., 2018, Nature Communications with permission, published under the Creative Commons Attribution 4.0 International Public License (CC BY 4.0; https://creativecommons.org/licenses/by/4.0/).

Single-molecule FRET (smFRET) approaches have many advantages as a structural biology method, including:

- sensitivity to macro-molecular distances (2.5–10 nm),
- the ability to resolve structural and dynamic heterogeneities,
- high-quality measurements with low sample consumption of the molecules of interest (low concentrations and low volumes), as the sample is analyzed one molecule at a time,
- determination of structural transitions in equilibrium, hence without the need for synchronization,
- the ability to detect (very) rare events. Indeed, in biology, the most interesting molecules to study are often the sparse, functionally active ones amidst a sea of inactive molecules,
- high sensitivity and specificity for labeled molecules. As only the labeled molecule uniquely contributes to the detected signal, these tracers can also be applied as FRET-reporters in crowded environments (Dupuis et al., 2014; Soranno et al., 2014; Zosel et al., 2020b) (hence smFRET can be used to validate results determined in isolation or detect the modulation of conformational preferences and/or structural dynamics through so-called quinary interactions [Guin and Gruebele, 2019]), and
- high specificity for residues/domains via specific labeling. Biomolecules can be specifically labeled by a unique dye pair enabling smFRET measurements to be applicable on all sizes of molecules, including large complex assemblies (see Figure 1 [Kilic et al., 2018]), active biological machines (e.g., the ribosomes) (Dunkle et al., 2011) and even on whole native virions (Lu et al., 2019; Munro et al., 2014).

Several methods have been utilized to determine structural ensembles such as NMR, single-particle cryoEM or XL-MS, and, recently, also smFRET in an integrative/hybrid (I/H) approach with computational modeling to overcome the sparsity of experimental data with respect to an atomistic description (Berman et al., 2019; de Souza and Picotti, 2020; Dimura et al., 2020; Gauto et al., 2019; Koukos and Bonvin, 2020; Na and Paek, 2020; Tang and Gong, 2020; Webb et al., 2018). I/H structural models derived from smFRET experiments using inter-dye distances as restraints were reported for flexible folded proteins (Brunger et al., 2011; Hellenkamp et al., 2017; Margittai et al., 2003; McCann et al., 2012), conformational ensembles of disordered/unstructured and unfolded proteins (Borgia et al., 2018; Holmstrom et al., 2018; Schuler et al., 2020), nucleic acids and protein-nucleic acid complexes (Craggs et al., 2019; Craggs and Kapanidis, 2012; Kalinin et al., 2012; Lerner et al., 2018b; Muschielok et al., 2008; Wozniak et al., 2008).

A further unique aspect of smFRET studies is that structural, kinetic, and spectroscopic information on large and complex systems can be recorded simultaneously in a single measurement. This facilitates linking dynamic and structural information in an integrative approach to (Figure 1A) (Hellenkamp et al., 2017; Kilic et al., 2018; Li et al., 2020b; Sanabria et al., 2020; Wasserman et al., 2016; Yanez Orozco et al., 2018):

- define the number of possible structures consistent with data,
- potentially reduce the ambiguity between different structural models compatible with the experimental data, and
- reveal the dynamic exchange pathways that are structurally allowed.

As an example, Figure 1B shows the outcome of a multimodal smFRET study on the conformational landscape of a 12-mer chromatin array (~2.5 MDa) (Kilic et al., 2018) with dynamics occurring on timescales from nanoseconds to hours. SmFRET experiments could detect the flexible chromatin conformations (Figure 1B, middle panel), revealing their dynamic structural heterogeneity (Figure 1B, bottom panel), in contrast to the well-ordered static structures of chromatin fibers (Figure 1B, top panel). These flexible, partially-open and open conformations that are quite abundant in solution (population of >70%; Figure 1B, bottom panel) were not resolved before, although they are essential for proper gene organization and function. They represent the central interconversion hub for the distinct stacking registers of chromatin and are difficult to detect with other structural techniques. This approach of visualizing biomolecules in action under ambient conditions emphasizes the importance of their dynamic nature by resolving transitions between various conformational states, which, in many cases, promotes function (Aviram et al., 2018; Henzler-Wildman et al., 2007; Iljina et al., 2020; Lerner et al., 2018b; Sanabria et al., 2020; Tassis et al., 2020).

SmFRET measurements are typically performed using two approaches: with surface-immobilized molecules using total internal reflection fluorescence microscopy (TIRFM) and camera-based detection, or with freely diffusing molecules in solution using confocal microscopy and point detectors. Experimental systems are available commercially but are typically home-built. Samples are prepared and the data collected using lab-specific protocols, where data are stored in a variety of file formats and analyzed using an array of increasingly powerful software. For the field in general and for structural studies in particular, it is important to demonstrate that smFRET, as a method, is reproducible and reliable regardless of where and how the sample is measured. To this end, in an effort led by Thorsten Hugel, twenty laboratories joined in measuring smFRET on several dsDNA constructs (Hellenkamp et al., 2018a). Studying six distinct samples with different dyes and varying inter-dye distances, the mean FRET efficiencies obtained by the participating labs exhibited a surprisingly high degree of agreement (a ΔE between 0.02 and 0.05 depending on the details of the sample). The quantitative assessment and reproducibility of the intensity-based smFRET measurements and discussions about data analysis was an important milestone. These dsDNA FRET standards are now available for every day calibration and are especially useful for new groups joining the community.

Encouraged by the insights gained in the above-mentioned FRET endeavor (Hellenkamp et al., 2018a), new multi-lab blind studies have been initiated. The next comparative FRET study, led by Thorben Cordes, investigates the robustness and reliability of smFRET experiments on proteins undergoing ligand-induced conformational changes (Gebhardt et al., in preparation). This study uses two distinct model proteins to assess the reproducibility and accuracy of protein-based smFRET for inter-dye distance determination measurements. Protein systems bring new challenges, including statistical dye labeling, site-specific dye properties, protein stability, shipping, storage and conformational dynamics. Hence, the study also assesses the ability of smFRET to discover and quantify dynamics on different timescales from microseconds to seconds. Another FRET challenge, initiated by Sonja Schmid, is the kinSoftChallenge (http://www.kinsoftchallenge.com, Götz et al., in preparation), which evaluates existing tools for extracting kinetic information from single-molecule time trajectories. This challenge aims to: (1) demonstrate the ability of smFRET-based kinetic analyses to accurately infer dynamic information and (2) provide the community with the means of evaluating the different available software tools.

One important outcome of the various multi-lab FRET studies was that, although the agreement was good, it could be improved even further. In particular, the data analysis, and specifically corrections, can have an impact on the determined FRET efficiencies and resulting distances. Hence, an open discussion regarding which approaches work most reliably under what conditions is necessary. Access to the primary data and the ability to process them with various analysis approaches is, and will remain, the most transparent way to move the field forward. Currently, this is difficult given the many variations in methods employed, their documentation, file formats and experimental procedures implemented across laboratories establishing the optimal conditions, workflow and best practices even for existing, well-tested methods is challenging since a comparison of these methods is time-consuming and the necessary information is, in many cases, not available. With the increase in open scientific practices and submission of published data to repositories, a consensus is needed regarding what data and metadata should be stored and in which possible formats so that it can be readily utilized by the community.

Due to these considerations and the many opportunities for growth of the smFRET community, several laboratories with expertise in FRET, without pretension to be exhaustive or exclusive, have gathered to endorse these efforts and propose steps to organize the community around consistent and open-science practices. This action translates into general methodological recommendations or suggestions, which we introduce following the typical workflow of a smFRET experiment, including sample preparation and characterization, setup description, data acquisition and preservation, and data analysis. These recommendations on how to ‘practice’ smFRET are *not* an attempt to regiment the community but rather an initial suggestion that aims at encouraging an open dialog about existing practices in our field and leads to higher reproducibility in the results from smFRET experiments. We then discuss open science practices as well as the first steps that have been taken to form an international FRET community. We end with highlighting a few of the areas where we see smFRET making a big impact in various scientific fields in the near future.

## State of the art of single-molecule FRET experiments

Within the FRET community, considerable know-how and expertise exists for the design, measurement and analysis of FRET experiments. In this section of the paper, we:

- review the workflow of smFRET experiments,
- discuss practical problems and potential pitfalls,
- provide recommendations for good practice, and
- list key scientific challenges that the field faces.

In the following, we consider each of these four aspects at every step of the smFRET workflow, from the choice of instrumentation all the way to the generation of structural and dynamic models.

### Experimental approaches: free diffusion or surface immobilization?

**Figure 2.**
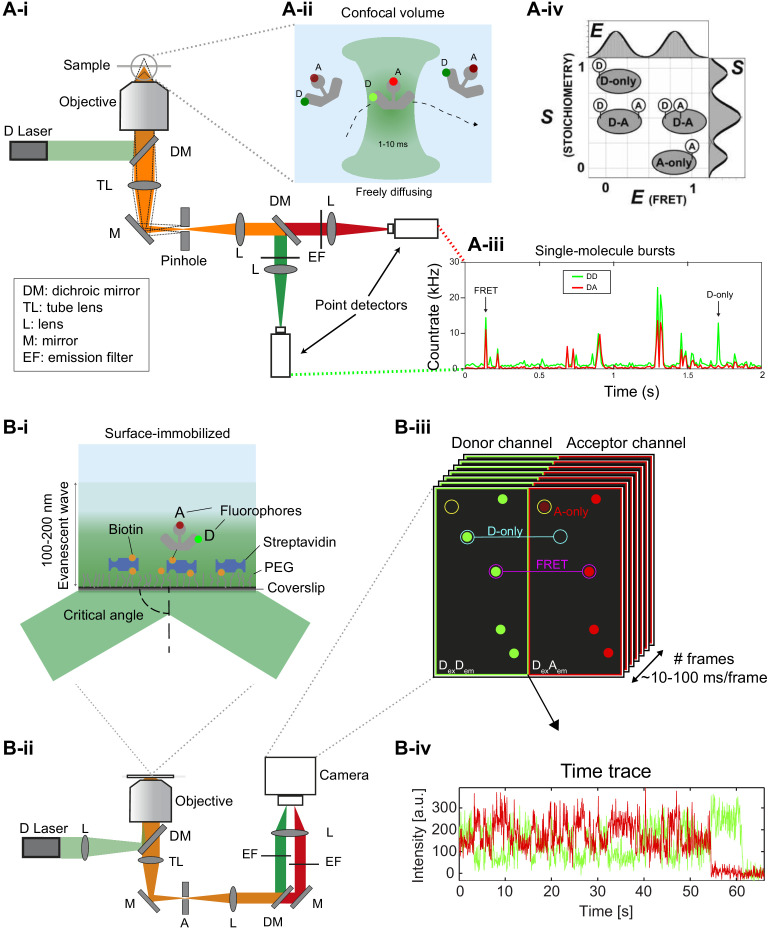
Different smFRET modalities. (**A**) Confocal smFRET measurements on freely-diffusing molecules. (i) A schematic of a single-color excitation confocal microscope with point detectors used for two-color detection. The excitation light is guided to the microscope body and reflected by a dichroic mirror (DM) toward a high numerical aperture (NA) objective lens that focuses the light in solution. The fluorescence emission is collected through the same objective lens, passes through the DM and pinhole and is spectrally split into donor and acceptor detection channels by a second DM in the detection path. After passing through emission filters (EF), single photons are detected on point detectors with high quantum efficiency, typically avalanche photodiodes (APD). (ii) Illustration of a double-labeled molecule freely diffusing through the confocal excitation spot. (iii) Exemplary confocal smFRET measurement showing photon bursts arising from single-molecules diffusing through the confocal volume. Green: Donor emission. Red: Acceptor emission. Exemplary bursts belonging to a single- or a double-labeled molecule are indicated with arrows. (iv) In ALEX or PIE experiments, the two-dimensional histogram of the molecule-wise FRET efficiency E and stoichiometry *S* allows one to separate single- and double-labeled populations (2005 Elsevier Ltd. All rights reserved. The figure was originally published as Figure 2A in Lee et al., 2005. Biophysical Journal, 88(4): 2939–2953. Further reproduction of this panel would need permission from the copyright holder). (**B**) TIRF-based smFRET experiments on surface-immobilized molecules. (i) Illustration of a surface-immobilized sample labeled with donor and acceptor fluorophores. (ii) Scheme of a single-color objective-type TIRF excitation two-color wide-field detection microscope. A: Aperture, TL: Tube lens, L: Lens, M: Mirror, DM: Dichroic mirror, EF: Emission filter. (iii) Illustration of an image of single molecules, in which the donor and acceptor (FRET) signals are split onto two halves of the camera. Mapping between the two channels is typically done using fluorescent beads (Joo and Ha, 2012; Roy et al., 2008; Zhuang et al., 2000) or zero-mode waveguides (Salem et al., 2019). (iv) Single-molecule fluorescence trajectory of the donor and acceptor (FRET) dyes, illustrating an anti-correlation indicative of FRET dynamics.

The workflow of smFRET studies starts with choosing one of the two most popular smFRET implementations: confocal and TIRF microscopy. Confocal microscopy is especially well-suited for studying freely diffusing molecules (Figure 2A), while TIRF microscopy is typically used for surface-immobilized molecules (Figure 2B; e.g., reviewed in Juette et al., 2014; Roy et al., 2008; Sasmal et al., 2016).

Compared to most other single-molecule approaches, both smFRET modalities offer relatively high throughput.

- In the confocal modality, the free diffusion of molecules into the observation volume and the short residence times enable the acquisition of many single-molecule events for extended amounts of time at rates of a few events per second. It can offer sub-nanosecond time resolution, yet single molecules are only observed during diffusion through the confocal excitation volume (typically <10 milliseconds). This allows one to obtain snapshots of thousands of individual molecules over the course of hours.
- In the TIRF modality, hundreds to thousands of dye-labeled molecules can be imaged simultaneously in one field of view. This approach reveals ‘motion pictures’ of individual molecules from seconds to minutes until the fluorophores photobleach. It typically has a lower temporal resolution of about a few tens of milliseconds but this is improving with technological advances. TIRF can be performed by illuminating through a high-numerical-aperture objective (Figure 2B) or through a quartz prism (Roy et al., 2008).

**Figure 3.**
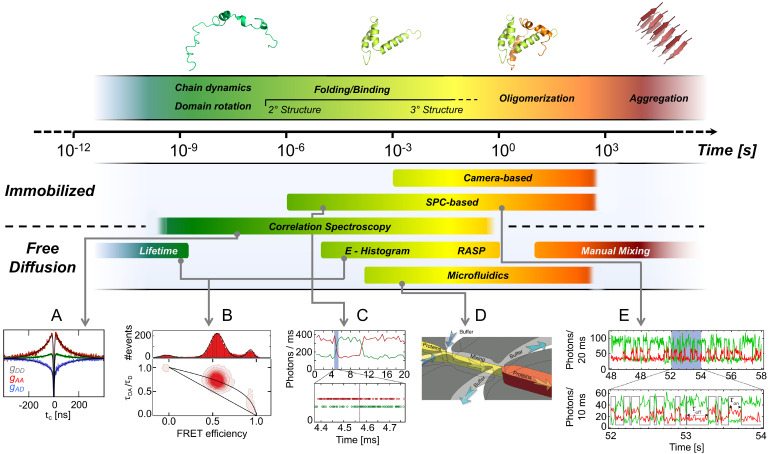
Exemplary methods for following smFRET dynamics on different timescales. Top: Biomolecular dynamics cover a wide range of timescales. Biomolecular rotations occur in the pico- to nanosecond range, while conformational changes take place in nano- to microseconds (ns-µs), as in chain dynamics of disordered proteins, and protein folding in microseconds to minutes. Transitions along energetically unfavorable pathways can take up to hours or longer, as in protein misfolding (Borgia et al., 2011; Tosatto et al., 2015). (2013 Elsevier Ltd. All rights reserved. The figure was originally published as Figure 1 in Schuler and Hofmann, 2013. *Current Opinion in Structural Biology*, 23(1): 36–47. Further reproduction of this panel would need permission from the copyright holder.) Bottom: (**A**) Picosecond (ps) to millisecond (ms) processes are typically examined with confocal methods such as polarization-resolved fluorescence lifetime measurements and Fluorescence Correlation Spectroscopy (FCS). Example shown: chain dynamics of an IDP from nsFCS. (**B**) Conformational states are identified by individual populations with characteristic positions in the FRET efficiency - lifetime diagrams as discussed in the sections Detection and characterization of intra-state dynamics and Future of smFRET (adapted from Soranno et al., 2012). (**C**) Fast transitions measured using confocal microscopy can be analyzed using the photon trajectory and applying a photon-by-photon maximum likelihood approach (2018 Elsevier Ltd. All rights reserved. The figure was originally published as Figures 2 and 3 in Chung and Eaton, 2018. *Current Opinion in Structural Biology*, 48: 30–39. Further adaptation of this panel would need permission from the copyright holder.) The timescale over which kinetics can be measured can be extended for diffusing molecules at low concentrations by using a recurrence analysis of single particles (RASP, Hoffmann et al., 2011). (**D**) Non-equilibrium experiments over extended periods of time can be performed with microfluidic mixing devices. (Copyright 2011, Nature Publishing Group, a division of Macmillan Publishers Limited. All Rights Reserved. Reproduced from Gambin et al., 2011, with permission. Nature Methods 8:239–241. Further reproduction of this panel would need permission from the copyright holder.) (**E**) Slow changes in conformations over a broad range of timescales can be followed in smFRET efficiency trajectories registered by single-photon counting (SPC) or cameras over minutes to many hours when the sample is immobilized (adapted from Figure 1 of Zosel et al., 2018).

When embarking on the investigation of conformational dynamics of a new biological system, the method of choice most often depends on the availability of the proper instrumentation. However, the dynamical aspects (reviewed in section Conformational dynamics) of the biological system under investigation, which are typically not known a priori, will eventually define which of the two methods is best suited. Because the dynamics of biological systems occur over a range of timescales from nanoseconds to seconds (Figure 3), ideally one would like to apply both modalities in parallel to obtain a complete understanding of the system (e.g., as shown in Figure 1).

Many variations exist with respect to the above-mentioned basic modalities to:

1) maximize the information content of the fluorescence signal.

- The confocal modality equipped with TCSPC and polarization-sensitive detections, so-called multiparameter fluorescence detection (MFD), allows monitoring of the fluorescence lifetime and anisotropy in addition to the fluorescence intensity (Kühnemuth and Seidel, 2001; Rothwell et al., 2003; Sisamakis et al., 2010; Widengren et al., 2006). The simultaneous collection and analysis of multiple parameters provides valuable insights into conformational dynamics, impurities and other spurious fluorophore-related artifacts.
- Alternating laser excitation (ALEX) (Kapanidis et al., 2004) allows for optical sorting of molecules exhibiting fluorescence from a single dye or from the two dyes in the FRET experiment (Figure 2A-iv) and also extract information on dye photophysics. In the TIRF modality, millisecond ALEX (msALEX) (Margeat et al., 2006) is typically used; in the confocal modality microsecond ALEX (μsALEX) (Kapanidis et al., 2005; Kapanidis et al., 2004; Lee et al., 2005) or nanosecond ALEX (nsALEX), aka. pulsed interleaved excitation (PIE) (Kudryavtsev et al., 2012; Laurence et al., 2005; Müller et al., 2005) are used.
- Three or more spectral channels can be used for multi-color smFRET (Clamme and Deniz, 2005; Hohng et al., 2004; Lee et al., 2010c; Lee et al., 2007a; Ratzke et al., 2014; Stein et al., 2011).

2) optimize data collection.

- A confocal microscope equipped with a laser and a sample or laser scanning module is also suited to study immobilized molecules (Chung et al., 2012; Edman et al., 1999; Ha et al., 1999; Ha et al., 1997; Hanson et al., 2007; Rhoades et al., 2003; Sabanayagam et al., 2004; Sturzenegger et al., 2018; Uphoff et al., 2011; Wang and Lu, 2010). It is the ‘best of both worlds’ in terms of timing, that is high time resolution and long observation times. However, it requires localizing and measuring each molecule individually, leading to lower throughput.
- Multi-spot detection, on arrays of single-photon avalanche diode detectors (SPAD arrays) and other state-of-the-art detectors, increases the throughput of confocal-based smFRET measurements and enables the study of non-equilibrium kinetics with higher time resolution (Ingargiola et al., 2016b; Ingargiola et al., 2018a; Segal et al., 2019).
- Objective-type TIRF can be combined with micro-mirrors in the excitation path to reduce background (Larson et al., 2014).
- Novel large-chip sCMOS cameras allow imaging at higher frame rates than their EMCCD counterparts. With the larger chip size, it can detect tens of thousands of molecules simultaneously (Juette et al., 2016) and the time resolution can be pushed into the sub-millisecond time scale (Fitzgerald et al., 2019; Girodat et al., 2020; Pati et al., 2020).

3) control the sample.

- In the confocal modality, the upper limit of the observation time can be pushed by recurrence analysis (Hoffmann et al., 2011) or by conjugating the molecules to large slowly-diffusing particles or liposomes (Diez et al., 2004; Kim et al., 2015a). Alternatively, the Moerner group confined molecules of interest to the observation volume without immobilization by using an anti-Brownian electrokinetic (ABEL) trap (Cohen and Moerner, 2005; Wilson and Wang, 2019).
- The space available for diffusion can be confined by using nanochannel devices (Fontana et al., 2019; Tyagi et al., 2014) or limiting the sectioning of the excited region through highly inclined and laminated optical (HILO) excitation (Gilboa et al., 2019) so that freely diffusing molecules can be tracked with camera detection.
- Microfluidics-based sample handling devices, including various mixers (Gambin et al., 2011; Hellenkamp et al., 2018b; Kim et al., 2011; Lemke et al., 2009; Lipman et al., 2003; Wunderlich et al., 2013; Zijlstra et al., 2017), allow automated sample handling and enable non-equilibrium measurements (Hamadani and Weiss, 2008; Juette et al., 2016).

The many possibilities available in the choice of hardware underscore the importance of precisely describing the components of the experimental setup. This includes optical elements (e.g., lenses, filters, mirrors, dichroics), light sources, optomechanical/optoelectronic devices and their characteristics, and detectors and their associated electronics. These details contribute in many ways to the finally recorded data and cannot, in general, be inferred retrospectively.

With the palette of FRET modalities increasing steadily, we recommend a rigorous comparative study of the different methods using well-characterized model samples. First and foremost, the study should determine the precision and limitations of each method and their complementarity. As one example, potential pitfalls in the determination of data correction factors (described in the section FRET efficiency) could be identified by a side-by-side comparison of fluorescence lifetime and intensity-based FRET methods.

### Sample preparation

#### Dyes

For studying biomolecular conformations and their dynamics with smFRET, the biomolecules of interest must be labeled with organic dyes that are suitable for single-molecule fluorescence detection (intrinsically fluorescent aromatic amino acids are not stable or bright enough). These dyes usually include three modules: (i) a chemically reactive group that forms a covalent bond preferentially with a specific nucleic acid base or amino acid residue of choice, (ii) a sufficiently long linker of a few connecting bonds to ensure isotropic rotation of the fluorophore, and (iii) an (often bulky) π-conjugated fluorophore that typically has hydrophobic regions and charged or polar substitutions.

To compete with background-noise, smFRET-compatible dyes should be very bright. They should hence possess a sufficiently large extinction coefficient (>50,000 M^−1^cm^−1^ at the wavelength of excitation) and high fluorescence quantum yield ($\phi_{F}$ ≳ 0.3), be very photostable (≳ 10^6^ excitation cycles before photobleaching), exhibit low photoblinking, should not possess long-lived dark states to avoid optical saturation and have a large fundamental anisotropy, that is have approximately collinear absorption and emission transition dipole moments (typically, $r_{0}$≳0.37). The fluorescence lifetime should be on the 1-5 ns scale. In the case of TCSPC experiments, a general rule of thumb is that the laser repetition period should be chosen at least four times as large as the fluorescence lifetime. For instance, for a dye with a fluorescence lifetime of 4 ns, a laser pulse repetition rate of ~64 MHz for one-color excitation or ~32 MHz for two-color nsALEX/PIE experiments should be used. In addition, using dyes with intrinsic mono-exponential fluorescence decays simplifies the analysis. Continuous efforts are ongoing to further improve smFRET dyes by:

- structural modifications of the core dye structure (Matikonda et al., 2020b): rhodamines and silicon rhodamines, carbopyronines, oxazines; cyanines (Matikonda et al., 2020a; Michie et al., 2017), carbocyanines; BODIPY dyes, perylenes or others, aiming to produce higher absorption cross-sections and fluorescence quantum yields (Grimm et al., 2017; Grimm et al., 2015), good chemical stabilities, water solubility (e.g., sulfonated carbocyanines) (Mujumdar et al., 1993) and a decoupling between the photophysical properties and the microenvironment (Hell et al., 2015; Levitus and Ranjit, 2011; Michie et al., 2017),
- ‘self-healing’ dyes, where the fluorophore is directly linked to a photostabilizing moiety to achieve high photon counting rates (Altman et al., 2012; Isselstein et al., 2020; Bodo et al., 1981; Pati et al., 2020; Schafer et al., 1982; van der Velde et al., 2013; Zheng et al., 2014),
- switchable, caged, and photoactivatable dyes for measuring multiple donor-acceptor distances (Jazi et al., 2017; Uphoff et al., 2010),
- using multiple acceptors, which can extend the overall duration of the fluorescence signal and/or the distance-range for FRET measurements (Krainer et al., 2015), and
- developing inorganic probes that are brighter or have long fluorescence lifetimes, such as nanoparticles and lanthanides, which have also been applied for FRET studies (Clegg, 1995; Guo et al., 2019; Léger et al., 2020).

Finally, a pair of FRET dyes should always be chosen such that its Förster distance, $R_{0}$, (defined in section Inter-dye distances, Equation 6) is around the expected inter-probe distance, $R_{DA}$, where the dependence of the FRET efficiency, E, is most sensitive to $R_{DA}$. When quantifying conformational dynamics, the FRET dye pair should be chosen such that the expected change in FRET efficiency is as large as possible.

#### Conjugation

To measure intra-molecular distances within biomolecules, smFRET experiments require the conjugation of two dye molecules to the same biomolecule or the same biomolecular complex. Site-specific conjugations in proteins utilize the introduction of point mutations, typically to cysteines, that will accommodate the specific conjugation chemistry, usually maleimide- or iodoacetamide-cysteine chemistry. In this case, two cysteines are often stochastically labeled, leading to a mixture of donor-acceptor and acceptor-donor labeled molecules. While interchanging the donor and acceptor positions has a negligible effect, from the geometric standpoint, on the FRET-averaged distance (Peulen et al., 2017), stochastic labeling might cause problems when the donor/acceptor dyes possess different spectroscopic properties at the different labeling positions.

Potential issues related to stochastic labeling can be excluded when, for example, a multi-dimensional analysis available from MFD-PIE shows no dye-induced sub-populations. Alternatively, stochastic labeling can also be avoided by:

- exploiting the differences in thiolate reactivities when carrying out double cysteine labeling (Hohlbein et al., 2013; Jacob et al., 2005; Orevi et al., 2014; Santoso et al., 2010a), or blocking the accessibility of specific cysteines (Jäger et al., 2005),
- combining cysteine labeling with bio-orthogonal labeling approaches such as unnatural amino acids (Chakraborty et al., 2012; Milles et al., 2012; Quast et al., 2019; Sadoine et al., 2017; Sanabria et al., 2020), native chemical ligation (Deniz et al., 2000), or using other bio-conjugation approaches that are specific and selective to other amino acids, for instance, methionine (Kim et al., 2020),
- purifying specific dye-labeled species via analytical chromatography (Lerner et al., 2013; Orevi et al., 2014; Zosel et al., 2020a),
- using different dyes that can be introduced to the same system using DNA hybridization (Auer et al., 2017; Deußner-Helfmann et al., 2018; Filius et al., 2020),
- the aid of self-labeling enzymes or peptide tags, such as SNAP-tag (Olofsson et al., 2014), HaloTag (Okamoto et al., 2020), ACP-tag (Meyer et al., 2006a; Meyer et al., 2006b; Munro et al., 2014; Wang et al., 2012), or the enzymes sortase (Kim and Chung, 2020) and transglutaminase (Jäger et al., 2006), and
- the use of fluorescent proteins (Düser et al., 2008; Okamoto et al., 2020), which have also been applied in smFRET studies.

Different approaches are applied for nucleic acids (e.g., reviewed in Hanspach et al., 2019; Steffen et al., 2019). For short nucleic acids, site-specific conjugation is generally achieved by post-synthetic labeling of reactive groups (e.g., through click chemistry) that are incorporated during solid-phase synthesis. Strategies have also been developed to site-specifically label longer RNAs (Anhäuser and Rentmeister, 2017; Baum and Silverman, 2007; Büttner et al., 2014; Zhao et al., 2018), and the use of hybridizing probes (Steiner et al., 2008) and fluorescent nucleobase analogues as intrinsic probes (Karimi et al., 2020; Steinmetzger et al., 2020) has been explored.

A general recommendation for labeling is to aim for high-purity sample preparations with optimized labeling protocols, as only this will result in substantially and specifically labeled samples with both donor and acceptor dyes. Single-molecule measurements have the ability to separate out the donor-acceptor-labeled molecules and thus purify the sample *ex post facto*, but a significant amount of double-labeled samples is advantageous. After labeling, we recommend using a rigorous screening procedure that compares the activities of labeled and unlabeled wild-type biomolecules to determine whether the mutations introduced to a biomolecule and/or the labeling with the dyes significantly influence the biomolecule's functionality (e.g., catalytic activity, binding affinity) and stability (e.g., against denaturants or thermally-induced transition curves) (Best et al., 2018; Deniz et al., 2000; Lerner et al., 2018b; Orevi et al., 2014; Riback et al., 2019; Sottini et al., 2020). To check for structural integrity, methods such as mass spectrometry, circular dichroism (CD), dynamic light scattering (DLS), and small-angle X-ray scattering (SAXS) can be used (Best et al., 2018; Borgia et al., 2016; Riback et al., 2019). We also recommend reporting the labeling and purification procedures as well as the labeling efficiency. In cases where no labeling alternative exists that does not modify the structure and/or rate of function, mechanistic insights into biomolecules or complexes can often still be obtained. Nevertheless, the results and conclusions concerning wild-type and unlabeled protein, respectively, should be interpreted cautiously. Finally, when samples need to be frozen/thawed, we recommend testing the long-term stability and functionality versus fresh protein preparations.

#### Immobilization

For long observation times, labeled molecules are typically immobilized. This is most frequently achieved via a biotin-streptavidin linkage. Immobilization must be carefully performed in order to systematically eliminate spurious contributions from molecules that are non-specifically bound (Lamichhane et al., 2010; Traeger and Schwartz, 2017). To address this potential issue, efforts have been made to optimize surface passivation procedures (Hua et al., 2014; Kuzmenkina et al., 2005; Park et al., 2020; Selvin and Ha, 2008). Alternatives that avoid the direct linking of biomolecules to surfaces are:

- mimicking a native environment by reconstitution of membrane proteins in nanodiscs (Bavishi et al., 2018; Hartmann et al., 2015) or liposomes (Diez et al., 2004),
- encapsulating biomolecules in spatially-restricted volumes such as liposomes (Boukobza et al., 2001; Cisse et al., 2007; Fitzgerald et al., 2019; Okumus et al., 2004; Rhoades et al., 2003; Zelger-Paulus et al., 2020). Care should be taken since the fraction of functioning proteins can be reduced due to the encapsulation process itself. Also, interactions between the protein and/or dyes and the lipids can pose a problem, and 
- precise positioning of biomolecular assemblies on DNA-origami platforms (Bartnik et al., 2020; Gietl et al., 2012).

We recommend reporting the immobilization conditions, the control experiments that demonstrate the specific nature of the surface immobilization strategy, and the percentage of functional or dynamic molecules (Bavishi and Hatzakis, 2014; Lamichhane et al., 2010; Roy et al., 2008) in detail. Finally, when possible, we recommend cross-validating the results of surface-immobilization based smFRET experiments by comparing them either to those obtained in ensemble or single-molecule FRET experiments on non-immobilized, freely-diffusing molecules (Pirchi et al., 2011), or to results using different immobilization strategies (Gregorio et al., 2017; Whitford et al., 2010).

#### Spectroscopic characterization

Fluorescent dyes are characterized by particular spectroscopic properties, which may change when conjugated to a protein (Lerner et al., 2013; Peulen et al., 2017; Sindbert et al., 2011; Steffen et al., 2016) or even between different structural states of the labeled biomolecule (Kudryavtsev et al., 2012). The most important artifacts to look out for are:

- photoblinking, photobleaching, changes of fluorescence anisotropies or the molecular brightness, and spectral shifts can create artifactual FRET-species when not properly identified and corrected for or removed (Chung et al., 2009; Kong et al., 2007; Sindbert et al., 2011; van der Velde et al., 2016). Protein-induced fluorescence enhancement (PIFE) (Hwang et al., 2011; Hwang and Myong, 2014) has to be taken into account for the donor properties and at the same time can serve as a molecular ruler at molecular distances inaccessible to other spectroscopic rulers in addition to FRET (Lerner et al., 2016; Ploetz et al., 2016),
- optical saturation effects that reduce the overall observed dye brightness (Gregor et al., 2005; Nettels et al., 2015). Acceptors that have a strong tendency for triplet-state formation or photoisomerization are particularly susceptible to optical saturation,
- dye-dye interactions that may lead to artificial high-FRET states (Sánchez-Rico et al., 2017) or to quenchable FRET (Cordes et al., 2010), and
- interactions between the dye and the labeled molecule can lead to dye-stacking in a predefined orientation that modulates the orientational factor, *κ^2^* (e.g., Cy3 base stacking to 5’-end of DNA [Liu and Lilley, 2017; Ouellet et al., 2011; Sanborn et al., 2007]), or they can lead to quenching and shifts in the apparent transfer efficiency, for example, via photoinduced electron transfer (PET) to aromatic groups (Doose et al., 2009; Haenni et al., 2013).

When the local and/or global environment influences the photophysical properties of either the donor or the acceptor dyes differently, different subpopulations might appear (Kalinin et al., 2010a; Vandenberk et al., 2018). Depending on the research question at hand, these subpopulations per se may provide additional information beyond FRET (e.g., PIFE [Ploetz et al., 2016], PET [Doose et al., 2009], or quenchable FRET [Cordes et al., 2010]). In cases where accurate distance measurements are needed, properly designed control experiments of fluorescence lifetimes and anisotropies of single-label versions for both labeling positions and dyes can be used to detect and eventually correct these spectroscopic alterations a posteriori. In addition, dye-artifacts can be identified from the information provided by ALEX or PIE experiments (Kapanidis et al., 2004; Kudryavtsev et al., 2012), MFD-based detection (Hellenkamp et al., 2017; Rothwell et al., 2003) or analysis of the width of FRET efficiency distributions (Kalinin et al., 2010a; Nir et al., 2006). Note that the influence of dye photoblinking must be taken into account: (1) when determining the correction factors necessary for precise FRET efficiency measurements (see section Determining absolute FRET efficiencies from fluorescence intensities) or (2) in the donor fluorescence quantum yield, when accurate distance estimations are required, which, in turn, depends on a correct Förster distance, $R_{0}$ (defined in section Inter-dye distances, Equation 6).

When dye- and microenvironment- dependent influences exist, they can be characterized or taken into account by a careful choice of fluorophores and/or labeling locations or coarse-grained computer simulations (Peulen et al., 2017), or they can be ruled out completely by validating the observations with (an)other FRET pair(s) (Borgia et al., 2018; Borgia et al., 2016; de Boer et al., 2019b; Husada et al., 2018; Lerner et al., 2017; Vandenberk et al., 2018; Voelz et al., 2012) or switching fluorophore positions (Sanabria et al., 2020). How important a detailed spectroscopic analysis is, depends on the nature of the research question being addressed.

#### Photostabilization

Often, chemical photostabilizers are added to reduce oxidative photodamage by lowering the time spent in triplet or radical-ion dark states (Ha and Tinnefeld, 2012; Widengren et al., 2007). The choice of the photostabilizing agent is specific to the fluorophore used and finding the correct conditions for both the donor and acceptor fluorophores can be challenging. Commonly used photostabilizers for smFRET include 6-hydroxy-2,5,7,8-tetramethylchroman-2-carboxylic acid (Trolox) (Cordes et al., 2009; Dave et al., 2009; Rasnik et al., 2006; Vandenberk et al., 2018), n-propylgallate (Widengren et al., 2007), β-mercaptoethanol (Campos et al., 2011; Ha and Tinnefeld, 2012), ascorbic acid (Aitken et al., 2008; Gidi et al., 2020; Vogelsang et al., 2008; Widengren et al., 2007), linear polyenes (Pfiffi et al., 2010) and cyclopolyenes (Dave et al., 2009; Targowski et al., 1987; Widengren et al., 2007), methylviologen (Vogelsang et al., 2008) and a range of other compounds (Glembockyte et al., 2015; Isselstein et al., 2020). For optimal performance, reducing and oxidizing agents can be combined (Dave et al., 2009; Vogelsang et al., 2008). Fluorophore performance and photon budgets can be enhanced by removing oxygen from the buffer through oxygen scavenging systems such as glucose oxidase (Kim et al., 2002) or the PCA/PCD system (Aitken et al., 2008), in which case an exogenous triplet quencher, such as those mentioned above, is required to prevent long-lived dark states. In any case, we recommend verifying that the use of these photostabilization reagents does not interfere with the biological system under study. In the case of lipid bilayers, an influence of several of the commonly used photostabilization agents on membrane properties was observed (Alejo et al., 2013).

### Molecule identification and validation

After data collection in either confocal or TIRF modalities, the single-molecule fluorescent signal in the resulting time traces or videos must be identified and validated before further detailed analysis can be performed.

#### Identification

In the confocal modality, the raw ‘burst’ data includes a sequence of photon detection or arrival times from at least two detectors. The first step is to identify fluorescence bursts arising from single molecules from the background, commonly referred to as the ‘burst search’ (Figure 2A–iii). Various approaches have been described for the robust and accurate detection of single-molecule events (Enderlein et al., 1997; Fries et al., 1998; Nir et al., 2006; Schaffer et al., 1999; Sisamakis et al., 2010). After the burst search step, the identified single-molecule events are filtered based on the burst properties (e.g., burst size, duration or width, brightness, burst separation times, average fluorescence lifetime or quantities calculated from these burst parameters). The burst search and burst selection criteria have an impact on the resulting smFRET histograms. Hence, we recommend that the applied burst property thresholds and algorithms should be reported in detail when publishing the results, for example, in the methods section of papers but potentially also in analysis code repositories. Often, burst search parameters are chosen arbitrarily based on rules-of-thumb, standard lab practices or personal experience. However, the optimal burst search and parameters vary based on the experimental setup, dye choice and biomolecule of interest. For example, the detection threshold and applied sliding (smoothing) windows should be adapted based on the brightness of the fluorophores, the magnitude of the non-fluorescence background and diffusion time. We recommend establishing procedures to determine the optimal burst search and filtering/selection parameters.

In the TIRF modality, molecule identification and data extraction can be performed using various protocols (Börner et al., 2016; Holden et al., 2010; Juette et al., 2016; Preus et al., 2016). In brief, the molecules first need to be localized (often using spatial and temporal filtering to improve molecule identification) and then the fluorescence intensities of the donor and acceptor molecules extracted from the movie. The local background needs to be determined and then subtracted from the fluorescence intensities. Mapping is performed to identify the same molecule in the donor and acceptor detection channels. This procedure uses a reference measurement of fluorescent beads or zero-mode waveguides (Salem et al., 2019) or is done directly on samples where single molecules are spatially well separated. The outcome is a time series of donor and acceptor fluorescence intensities stored in a file that can be further visualized and processed using custom scripts. In a next step, filtering is generally performed to select molecules that exhibit only a single-step photobleaching event, that have an acceptor signal when the acceptor fluorophores are directly excited by a second laser, or that meet certain signal-to-noise ratio values. However, potential bias induced by such selection should be considered.

#### User bias

Despite the ability to manually determine burst search and selection criteria, molecule sorting algorithms in the confocal modality, such as those based on ALEX/PIE (Kapanidis et al., 2005; Kudryavtsev et al., 2012; Tomov et al., 2012), do not suffer from a substantial user bias. In the early days, many TIRF modality users have relied on visual inspection of individual single-molecule traces. Such user bias was considerably reduced by the use of hard selection criteria, such as intensity-based thresholds and single-step photobleaching, intensity-based automatic sorting algorithms (e.g., as implemented in the programs MASH-FRET [Hadzic et al., 2019], iSMS [Preus et al., 2015] or SPARTAN [Juette et al., 2016]), and, most recently, artificial intelligence-based molecular sorting (deepFRET [Thomsen et al., 2020] and AutoSiM [Li et al., 2020a]).

Single-molecule experiments are often advertised as being able to detect rare events. Nonetheless, even for such sparsely populated states, it has to be confirmed that they are biologically relevant and neither a result of the selection procedure, coincidence or photophysical artifacts. To this end, users should specify how selections were performed and what percentage of the molecules was used for further analysis.

Ideally, a recommended protocol with implicit validation would be to start in the confocal modality to determine (i) the degree of labeling, (ii) the FRET properties of major biochemical species, and (iii) their populations and dynamic properties (see Figure 1). With this information at hand, experiments can be performed in the TIRF modality, where the percentage of FRET-active molecules and their FRET properties can be directly compared with the confocal data. Both datasets should be mutually consistent and, in this way, provide direct feedback with respect to potential artifacts (e.g., due to immobilization).

### Conformational dynamics

Many users in the FRET community employ the detection and characterization of different subpopulations or measurements of conformational dynamics as a handle to study biomolecules or biomolecular systems. Conformational dynamics are typically defined as:

- conformational transitions between distinct states separated by an activation barrier, typically defined as larger than the thermal energy, $k_{B}T$, where $k_{B}$ is Boltzmann’s constant and T is the absolute temperature, and
- or conformational fluctuations within states, defined by the shape of the potential wells between activation barriers.

Transitions can occur under equilibrium conditions, can be induced by the addition of substrates, ligands, or interaction partners (de Boer et al., 2019a; Mapa et al., 2010; Mazal et al., 2018; Schluesche et al., 2007); induced by mixing with denaturants (Kuzmenkina et al., 2006; Lindhoud et al., 2015; Maity and Reddy, 2016; Moosa et al., 2018; Nienhaus, 2006; Pirchi et al., 2011; Rieger et al., 2011; Schuler et al., 2002); or triggered by temperature (Ebbinghaus et al., 2010; Holmstrom et al., 2014; Nettels et al., 2009; Zhao et al., 2010a) and pressure modulations (Schneider et al., 2018; Sung and Nesbitt, 2020). Structural transitions can also occur spontaneously.

SmFRET is unique in that it allows the detection and analysis of equilibrium and non-equilibrium conformational dynamics across at least 12 orders of magnitude in time, that is from the nanoseconds to, in principle, thousands of seconds (Figure 3). Notably, it is important to optimize the labeling positions to maximize the distinction between different conformational states based on their FRET efficiencies (Dimura et al., 2020).

#### Detecting dynamics

Biomolecules are dynamic systems that show conformational flexibility and dynamics on fast time scales (Henzler-Wildman and Kern, 2007). Oftentimes, conformational interconversions occur on a timescale faster than the sampling time of the detection system, for example < 10 ms for TIRF modality or < 0.1 ms for confocal modality, resulting in the observed single-molecule time series or FRET efficiency histogram exhibiting only time-averaged FRET values, weighted by the fractional population of each conformational state. Several groups have developed methods for detecting and analyzing such ‘dynamic averaging’ from confocal-modality data. In general, these methods allow retrieval of dynamics on the milliseconds and sub-millisecond timescales by analyzing the average fluorescence lifetimes and/or photon counting statistics of single-molecule bursts. The precise knowledge of the experimental shot noise separates smFRET from other techniques in structural biology and enables a quantitative analysis of fluctuations caused by biomolecular dynamics. A number of methods have been developed for detecting and quantifying smFRET dynamics, which we discuss in more detail below on slower (section Slow dynamics) and faster time scales (section Faster dynamics). The first step in analyzing smFRET dynamics is the verification that dynamics are present. Popular methods for the visual detection of dynamics include:

- 2D histograms of burst-integrated average donor fluorescence lifetimes versus burst-integrated FRET efficiencies (Gopich and Szabo, 2012; Kalinin et al., 2010b; Rothwell et al., 2003; Schuler et al., 2016),
- burst variance analysis (BVA) (Torella et al., 2011),
- two-channel kernel-based density distribution estimator (2CDE) (Tomov et al., 2012),
- FRET efficiency distribution-width analysis, for example by comparison to the shot noise limit (Antonik et al., 2006; Gopich and Szabo, 2005a; Ingargiola et al., 2018b; Laurence et al., 2005; Nir et al., 2006) or known standards (Geggier et al., 2010; Gregorio et al., 2017; Schuler et al., 2002), and time-window analysis (Chung et al., 2011; Kalinin et al., 2010a; Gopich and Szabo, 2007), and
- direct visualization of the FRET efficiency fluctuations in the trajectories (Campos et al., 2011; Diez et al., 2004; Margittai et al., 2003).

#### Slow dynamics

**Figure 4.**
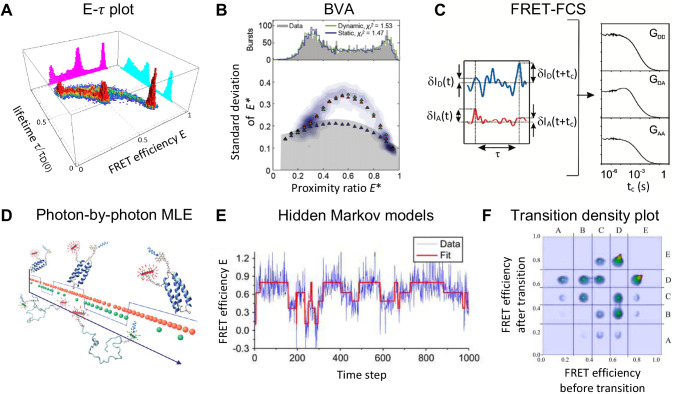
Exemplary selection of approaches to detect and quantify conformational dynamics in smFRET. (**A**) Dynamics in a three-state system are detected using the two-dimensional distribution of the FRET efficiency and donor fluorescence lifetime (Reproduced from Gopich and Szabo, 2012. Further reproduction of this panel would need permission from the copyright holder.) (**B**) The two-state dynamics of a DNA hairpin are revealed from the standard deviation of the proximity ratio $E^{*}$ that is higher than expected for photon counting statistics alone in the burst variance analysis (BVA). (2011 The Biophysical Society. Published by Elsevier Inc All rights reserved. The figure was originally published as Figure 4C in Torella et al., 2011. Biophysical Journal, 100(6): 1568–1577. Further reproduction of this panel would need permission from the copyright holder.) (**C**) In fluorescence correlation spectroscopy (FCS), the dynamics show up as a positive correlation in the autocorrelation functions $G_{DD}$ and $G_{AA}$ and an anti-correlation in the cross-correlation function $G_{DA}$ (2010 American Chemical Society Ltd. All rights reserved. The figure was originally published as Figure 1B and C in (Gurunathan and Levitus, 2010, reproduced with permission). Copyright 2010 ACS Publications. Further reproduction of this panel would need permission from the copyright holder.) (**D**) Photon-by-photon maximum likelihood estimation (MLE) infers the kinetic parameters directly from the photon arrival times (2011 American Chemical Society Ltd. All rights reserved. The figure was originally published as the Abstract Figure in Chung et al., 2011, reproduced with permission. Copyright 2011 ACS Publications. Further reproduction of this panel would need permission from the copyright holder.) (**E–F**) A hidden Markov model (HMM) is applied to the time traces of the FRET efficiency to estimate the states and interconversion rates (**E**). From the transition density plot (**F**), the connectivity of the FRET states is revealed. Displayed data in **E** and **F** are simulated. (Panels **E** and **F**: 2006 The Biophysical Society. Published by Elsevier Inc All rights reserved. The figures were originally published as Figure 4A and D in McKinney et al., 2006. *Biophysical Journal*, 91(5): 1941–1951. Further reproduction of this panel would need permission from the copyright holder.)

For dynamics on the order of 10 ms or slower, transitions between conformational states can be directly observed using TIRF-modality approaches, as have been demonstrated in numerous studies (Blanchard et al., 2004; Deniz, 2016; Juette et al., 2014; Robb et al., 2019; Sasmal et al., 2016; Zhuang et al., 2000). Nowadays, hidden Markov models (HMM) (Figure 4E) are routinely used for a quantitative analysis of smFRET time traces to determine the number of states, the connectivity between them and the individual transition rates (Andrec et al., 2003; Keller et al., 2014; McKinney et al., 2006; Munro et al., 2007; Steffen et al., 2020; Stella et al., 2018; Zarrabi et al., 2018). Below, we list extensions and other approaches for studying slow dynamics.

- Classical HMM analysis has been extended to Bayesian inference-based approaches such as variational Bayes (Bronson et al., 2009), empirical Bayes (van de Meent et al., 2014), combined with boot-strapping (Hadzic et al., 2018) or modified to infer transition rates that are much faster than the experimental acquisition rate (Kinz-Thompson and Gonzalez, 2018).
- Bayesian non-parametric approaches go beyond classical HMM analysis and also infer the number of states (Sgouralis et al., 2019; Sgouralis and Pressé, 2017).
- Hidden Markov modeling approaches have been extended to detect heterogeneous kinetics in smFRET data (Hon and Gonzalez, 2019; Schmid et al., 2016).
- Concatenation of time traces in combination with HMM can measure kinetic rate constants of conformational transitions that occur on timescales comparable to or longer than the measurement time (Kim et al., 2015b).
- In the confocal modality, slower timescales are accessible by exploiting the reentry of single molecules into the observation volume (recurrence analysis of single particles, RASP) (Hoffmann et al., 2011).

There are still many challenges with respect to the accuracy of the approaches that need to be discussed and improvements made to provide a reliable determination of kinetics.

#### Faster dynamics

Several methods exist that assist in the quantification of the kinetic parameters governing fast conformational dynamics, as also exemplified in Figure 3A,B.

- Dynamic photon distribution analyses (PDA) that analyze the width of FRET efficiency distributions with respect to photon shot noise and broadening by dynamic exchange (Gopich and Szabo, 2007; Kalinin et al., 2010b; Santoso et al., 2010b).
- Applying hidden Markov models on a photon-by-photon basis extends the achievable time resolution into the microsecond regime (Aviram et al., 2018; Keller et al., 2014; Mazal et al., 2019; Pirchi et al., 2016). More generally, photon-by-photon maximum likelihood analysis (Figure 4D) of diffusing or immobilized molecules has made it possible to extract sub-millisecond transition rates (Chung and Gopich, 2014; Chung et al., 2011; Gopich and Szabo, 2009), transition path times of protein folding (Chung et al., 2012; Chung and Eaton, 2018) and binding of disordered proteins (Kim et al., 2018a; Sturzenegger et al., 2018; Kim and Chung, 2020) on the microsecond timescale.
- Using confocal-modality approaches, numerous studies also directly mapped transitions between conformational states for dynamics on the order of 0.5 ms or slower (Diez et al., 2004; Hanson et al., 2007; Margittai et al., 2003).
- Plasmonic enhancement of the fluorescence signal, reaching count rates in the megahertz regime for single molecules, allows a direct visualization of dynamics on the sub-millisecond timescale without analysis of the photon statistics (Acuna et al., 2012; Bohlen et al., 2019).
- Higher time resolution for TIRF experiments below 10 ms can be achieved using stroboscopic illumination (Farooq and Hohlbein, 2015) or fast sCMOS cameras (Fitzgerald et al., 2019; Girodat et al., 2020; Juette et al., 2016; Pati et al., 2020), reaching into the sub-millisecond domain.

Fluorescence correlation spectroscopy (FCS) (Magde et al., 1972; Rigler et al., 1993) methods have also been widely applied and the observed kinetic rates are model-independent (i.e., unbiased).

- By combining smFRET with FCS (Felekyan et al., 2013; Gurunathan and Levitus, 2010; Margittai et al., 2003; Schuler, 2018; Torres and Levitus, 2007), it is possible to quantify FRET dynamics that are faster than the diffusion timescale (Figure 4C).
- FRET dynamics as fast as a few picoseconds can also be retrieved from a variant of FCS dubbed ‘*nanosecond FCS*’ (nsFCS) (Nettels et al., 2008; Nettels et al., 2007; Schuler and Hofmann, 2013; Figure 3A).
- Using statistical filters, it is possible to extract species-specific properties and to quantify the exchange rates between different sub-populations (filtered-FCS) (Böhmer et al., 2002; Enderlein et al., 2005; Felekyan et al., 2013; Kapusta et al., 2007).
- Species-specific hydrodynamic radii can be extracted using single-burst FCS (Bravo et al., 2018; Laurence et al., 2008; Laurence et al., 2007). In combination with FRET-FCS or filtered-FCS, this approach simplifies the analysis of kinetics by eliminating the contribution of single-labeled molecules (Barth et al., 2018; Felekyan et al., 2013).

In the analysis of fast dynamics and subpopulations, it is generally important to account for fast photophysical transitions, such as dye photoblinking, and for interactions of the dyes with the biomolecular surface, which may interfere with subpopulation dynamics and result in inaccurate transition rates (Chung and Gopich, 2014; Ingargiola et al., 2018b; Lerner et al., 2018b).

The detection and analysis of fast dynamics is one of the issues addressed in the protein FRET challenge (Gebhardt et al., in preparation). Finally, at the moment of this writing, different analysis algorithms are typically being applied to the data independently from each other (e.g., MFD, BVA, 2CDE, PDA, filtered-FCS in burst analysis) while, in fact, they could corroborate each other or even help in deciding on models. The field could focus on creating global multi-algorithm workflows or tools to test, how a model obtained with one algorithm would influence the results of other analyses.

#### Detection and characterization of intra-state dynamics

Rapid structural dynamics *within* a given conformational state, that is within a single energy minimum, can also be studied with smFRET by modeling them as a continuous distribution rather than a state-dependent distribution. In the given example, rigid and flexible conformational states can be distinguished in the measurement. Information regarding the flexibility of a given conformation can be retrieved by:

- describing their kinetic signatures in FRET efficiency vs. lifetime (E−τ) plots (Figure 4A–B) (Gopich and Szabo, 2012; Kalinin et al., 2010b),
- using the fluorescence lifetime information available with TCSPC, which can be used to analyze sub-ensemble fluorescence decays and retrieve the inter-dye distance distribution and the inter-dye distance diffusion coefficient (Gansen et al., 2018; Lerner et al., 2014; Neubauer et al., 2007; Rahamim et al., 2015; Sisamakis et al., 2010),
- analyzing brightness by sub-ensemble fluorescence intensity distribution analysis (FIDA) (Neubauer et al., 2007),
- analyzing the photon statistics of the time-stamped photon arrival trajectories (Ingargiola et al., 2018b; Ramanathan and Muñoz, 2015), and
- relating the time-averaged FRET efficiencies and subpopulation-specific nsFCS to polymer models or simulated ensembles (Borgia et al., 2018; Holmstrom et al., 2018).

It is, however, important to mention that the distinction between dynamics within a conformational state and dynamics of transitions between different conformational states is still under debate, which highly depends on the definition of an activation barrier for different modes of structural dynamics and on the different smFRET modalities used.

### FRET efficiency

The efficiency of the energy transfer process, that is the FRET efficiency, E, is defined as the fraction of donor excitations that result in energy transfer. Assuming a single distance between the centers of the donor and acceptor molecules, $R_{DA}$, the FRET efficiency is given by:

(1) $$E=\frac{k_{FRET}}{k_{FRET}+k_{D}}=\frac{1}{1+{\left(\frac{R_{DA}}{R_{0}}\right)}^{6}},$$

where $k_{FRET}$ is the rate of energy transfer, $k_{D}$ is the rate of donor de-excitation in the absence of an acceptor molecule, and $R_{0}$ is the Förster distance (discussed in section Dye models). Hence, FRET is indeed a tool that can measure distances on the molecular scale (Förster, 1948; Stryer and Haugland, 1967). For many smFRET studies, a qualitative indicator of the inter-probe distance is sufficient, for example, to merely be able to distinguish between conformational subpopulations or their transitions. Therefore, for all FRET experiments that do not require the exact inter-dye distance, the absolute value of E does not need to be known. However, special care should be taken to ensure that the observed changes of the donor and acceptor intensities report on a structural change of the molecule and are not a result of dye photophysics or dye-surface interactions. In the cases where accurate distance measurements are desired, smFRET can be used for that purpose.

#### Determining absolute FRET efficiencies from fluorescence intensities

Typically, in smFRET, the FRET efficiency is determined from the fluorescence intensities:

(2) $$E=\frac{F_{Aem|Dex}}{F_{Dem|Dex}+F_{Aem|Dex}},$$

where $F_{Aem|Dex}$ is the sensitized fluorescence signal from the acceptor after donor excitation and $F_{Dem|Dex}$ is the signal emanating from the donor. Here, we use a notation specific to experiments using alternating laser excitation, but equivalent expressions can be derived for single-color excitation. In reality, the absolute value for E requires knowledge of some correction factors (Hellenkamp et al., 2018a; Lee et al., 2005):

(3) $$E=\frac{\left[I_{Aem|Dex}-\alpha I_{Dem|Dex}-\delta I_{Aem|Aex}\right]}{\gamma \left[I_{Dem|Dex}\right]+\left[I_{Aem|Dex}-\alpha I_{Dem|Dex}-\delta I_{Aem|Aex}\right]},$$

where $I_{Aem|Dex}$ is the background-corrected signal in the acceptor emission channel after donor excitation, $I_{Dem|Dex}$ is the background-corrected signal in the donor emission channel after donor excitation and $I_{Aem|Aex}$ is the background-corrected signal in the acceptor emission channel after acceptor excitation. The last term can be estimated using the acceptor-only species and fluorescence signal after acceptor excitation when the ALEX/PIE method is used (Hellenkamp et al., 2018a; Kudryavtsev et al., 2012; Lee et al., 2005) or by comparing fluorescence intensities before and after donor photobleaching prior to acceptor photobleaching in trajectories from immobilized molecules (Yoo et al., 2018).

The required correction factors are:

- *α*, the fraction of the donor fluorescence signal detected in the acceptor channel due to spectral crosstalk,
- *δ*, the fraction of acceptor photons arising from excitation of the acceptor at the wavelength of the donor-exciting laser, directly, and not excitation via energy transfer,
- the *γ* factor (Ha et al., 1999), which compensates for the fact that the number of photons detected from the donor and acceptor fluorophores is not proportional to the number of their excitation/de-excitation cycles for two reasons: (i) fluorophores, in general, have different fluorescence quantum yields, $\phi_{F}$ values, and (ii) the efficiencies of detecting photons are different for the two channels due to different optical transmission efficiencies (owing to the characteristics of the filters and optics used) and different spectral sensitivities of the detectors.

The optimal procedures for determining the correction parameters is still a matter of active debate within the community. In the following, we focus on the *γ* factor, which we identify as the major contribution to uncertainty in smFRET experiments.

#### Determining the *γ* factor in confocal mode

Whenever a broad E distribution is reported in the confocal mode, the *γ* factor can be extracted using ALEX/PIE measurements. This method exploits the fact that the stoichiometry parameter, *S* (Kapanidis et al., 2004) (i.e., the ratio between the number of photons emitted after donor excitation and the number of photons emitted after donor and acceptor excitations), is independent of E when properly corrected for *γ*. It is thus essential that the sample contains two or more species with different distances and thus FRET efficiencies, E, yet identical values of *γ* for this method to work (Lee et al., 2005). Thus, accurate measurements of $\phi_{F}$ for both dyes have to be performed for each species. Alternatively, fluorescence lifetime measurements and the correlated analysis of intensity and lifetime data is often used to determine individual *γ* factors for each E sub-population, since lifetime-based FRET, in principle, provides the absolute E of a sub-population of single-molecule bursts independently from its intensity-based counterpart (Rothwell et al., 2003; Sisamakis et al., 2010; Vandenberk et al., 2018). However, when one or more species are dynamically averaged, a proper determination of the *γ* factor becomes more challenging and different assumptions need to be made.

Currently, the uncertainty in the determination of *γ* is one of the largest contributions to discrepancies of smFRET histograms measured from different laboratories (Gebhardt et al., in preparation). Hence, it would be beneficial to discuss optimal approaches to determine a robust confocal-mode *γ* value.

#### Determining the *γ* factor in TIRF mode

When ALEX data are collected on immobilized samples, the *γ* factor can also be estimated for individual molecules, provided that the acceptor photobleaches before the donor (Ha et al., 1999; Hildebrandt et al., 2015; McCann et al., 2010). Here, the decrease in the acceptor signal and the increase in donor signal upon acceptor photobleaching can be directly compared. This is also true for molecules undergoing slow dynamics between different conformations as the changes in intensity reflect the changes in detection efficiency. For this approach to be accurate, however, the acceptor must not enter a transient (e.g., redox or triplet) state that still absorbs energy from the donor (Hofkens et al., 2003; Nettels et al., 2015). The individual *γ* factors are usually broadly distributed, indicating a potential variability in its value. Nevertheless, an average *γ* factor is often applied to molecules where the donor photobleaches before the acceptor.

#### Determining absolute FRET efficiencies from fluorescence lifetimes

In addition to the traditional intensity-based FRET efficiency (E) can also be determined from the fluorescence lifetime ($\tau_{D(A)}$) of the donor in the presence and absence of the acceptor, denoted by $\tau_{D(A)}$ and $\tau_{D(0)}$, respectively. Assuming a single distance between donor and acceptor fluorophores (i.e., no distance fluctuations), the FRET efficiency is given by:

(4) $$E=1-\frac{\tau_{D(A)}}{\tau_{D(0)}}$$

The advantage of this approach is that correction factors are not needed, as most of the above-mentioned corrections influence the relative number of photons detected in the donor and acceptor channels, but not the donor fluorescence decay. The lifetime approach can also be used in ensemble/imaging measurements under conditions of incomplete labeling. Combined intensity- and lifetime-based FRET efficiencies can additionally be used for checking the self-consistency of the data and for detecting dynamics (e.g., via E-τ plots) (Gopich and Szabo, 2012; Kalinin et al., 2010b; Rothwell et al., 2003; Schuler et al., 2016).

#### Other methods for determining FRET efficiencies

There are additional procedures for determining the FRET efficiency, most of which are compatible with single-molecule fluorescence techniques. The FRET efficiency can also be determined:

- from the steady-state donor anisotropy (Clegg, 1992),
- from the ratio of the acceptor’s intensity after donor excitation to the acceptor’s intensity after acceptor excitation (Clegg, 1992),
- from the acceptor’s intensity in the presence and absence of the donor (e.g., via donor photobleaching) (Clegg et al., 1992),
- from the donor’s intensity in the presence and absence of the acceptor (e.g., via acceptor photobleaching) (Bastiaens et al., 1996),
- from time-resolved anisotropy measurements, in particular in homo-FRET experiments, where two identical probes are used as a donor-acceptor pair (Bergström et al., 1999; Somssich et al., 2015),
- using fluorescence correlation spectroscopy methods (Müller et al., 2005; Widengren et al., 2001).

### Inter-dye distances

When smFRET experiments are used for structural studies or accurate distance determination is desired, many steps need to be taken to convert the raw data (photons detected and registered by the detectors) into absolute inter-dye distances. In essence, it requires exact knowledge of the Förster distance, $R_{0}$ (also referred to as the Förster radius) and therefore of all parameters required for determining it, as well as knowledge with respect to the flexibility of the attached fluorophores (approximated using a dye-model). In this section, we review the various issues involved.

#### Förster distance $R_{0}$

In FRET, the excitation energy of the donor fluorophore is transferred to an acceptor fluorophore via weak dipolar coupling. Considering a single donor-acceptor distance, $R_{DA}$, the efficiency, E, of this non-radiative transfer process scales with the sixth power of $R_{DA}$ normalized by the Förster distance, $R_{0}$ (Equation 1). In smFRET studies, dyes are usually coupled to the biomolecules via long (ranging typically between 10 and 15 atoms) mostly flexible linkers, which result in an equilibrium distribution of $R_{DA}$ values, $p(R_{DA})$, caused by the flexibility of the dye linkers. In this case, one may observe a mean FRET efficiency ⟨E⟩ related to the FRET efficiency, averaged over all distances and their probabilities:

(5) $$\left\langle E\right\rangle =\int_{0}^{\infty }\frac{p\left(R_{DA}\right)}{1+{\left(\frac{R_{DA}}{R_{0}}\right)}^{6}}dR_{DA}.$$

It is noteworthy to mention that Equation 5 holds under the assumption that the inter-dye distance remains unchanged during the excited-state lifetime of the donor fluorophore. From the mean FRET efficiency ⟨E⟩, one obtains the FRET-averaged apparent donor-acceptor distance, ${\left\langle R_{DA}\right\rangle }_{E}$, which differs from the distance between the mean dye positions (Kalinin et al., 2012) and is dependent on the flexibility and dynamics of the dye.

As mentioned before, $R_{0}$ (Equation 1) is the distance at which half of the donor de-excitation events occur via energy transfer to the acceptor fluorophore. $R_{0}$ (in Å) is given by:

(6) $$R_{0}=0.2108{\left(\frac{\kappa^{2}\Phi_{F,D\left(0\right)}}{n_{im}^{4}}\int {\overset{-}{F}}_{D}\left(\lambda \right)\varepsilon_{A}\left(\lambda \right)\lambda^{4}d\lambda \right)}^{\frac{1}{6}},$$

meaning that it depends on the donor fluorescence quantum yield in the absence of an acceptor, $\phi_{F,D\left(0\right)}$, the overlap between the area-normalized donor emission spectrum, ${\overset{-}{F}}_{D}(\lambda )$, and the acceptor excitation spectrum with extinction coefficient, $\varepsilon_{A}(\lambda )$ (in *M^−1^cm^−1^*), at the wavelength λ (in nm), the relative orientation of the dye dipoles captured by the orientation factor, *κ^2^*, and the refractive index of the medium, $n_{im}$, between and around the dyes. It should be noted that, due to the $\lambda^{4}$ dependence of the overlap integral, small shifts in the spectra can have large effects on the $R_{0}$. The following sections describe the factors that influence $R_{0}$ and the FRET efficiency in more detail.

#### Extinction coefficient *ε*

The extinction coefficient of the acceptor dye affects $R_{0}$ and the expected excitation rate in ALEX/PIE experiments. In the absence of an easy or affordable way to measure this parameter (it requires large amounts of dye for gravimetric analysis or FCS with controlled dilution [Fries et al., 1998]), the experimenter typically relies on the value given by the manufacturer, a value that can at times be unreliable. Alternatively, the extinction coefficient of the dyes may be theoretically assessed via the Strickler and Berg, 1962 equation, when $\phi_{F,D(0)}$ and the fluorescence lifetime are known. Fortunately, *ε* is not expected to vary much depending on the environment of the fluorophores, since both the $\phi_{F,D(0)}$ and the fluorescence lifetime, in most cases, vary accordingly. Hence, one can conclude that the local environment does not heavily influence the excitation probability (according to the Strickler-Berg equation mentioned above).

#### Fluorescence quantum yield $\phi_{F}$

$\phi_{F}$ oftentimes changes upon labeling and can be sensitive to the local environment at the labeling position, to the conformational state of the molecule and to the binding of ligands, substrates or complex partners. Even dyes that are considered relatively insensitive to their local environment have been shown to exhibit a large change in $\phi_{F}$ upon conjugation to nucleic acids or proteins. As an extreme example, the quantum yield of Cy3B ranges from 0.19 to 0.97 at different labeling positions on dsDNA, leading to considerable variation in the value of $R_{0}$ for the pair Cy3B-ATTO 647N between 54.8 Å and 65.9 Å (Lerner et al., 2018b; Craggs et al., 2019). For dyes of the cyanine family, such as Cy3 and Cy5, or its variants Alexa Fluor 555 and Alexa Fluor 647 (Gebhardt et al., in preparation), $\phi_{F}$ is dependent on the excited-state isomerization, which is influenced by viscosity, steric restriction and (stacking) interactions (Hwang and Myong, 2014; Lerner et al., 2016; Levitus and Ranjit, 2011; Sanborn et al., 2007; White et al., 2006; Widengren et al., 2001). In summary, independent determination of $\phi_{F}$ for different labeling positions is strongly recommended. Notably, nsALEX/PIE and MFD experiments can probe the fluorescence lifetime, and thus directly identify changes in $\phi_{F}$. Development of standard procedures for measuring or estimating $\phi_{F}$, for example using an integrating sphere (Gaigalas and Wang, 2008; Pati et al., 2020) or a nanocavity (Chizhik et al., 2013; Chizhik et al., 2011), would benefit the field and should be discussed.

#### Refractive index $n_{im}$

The actual index of refraction to be used for calculation of $R_{0}$ lies somewhere between the index of refraction of an aqueous buffer (1.33) and that for proteins and DNA (~1.5) but the exact value is not known. Robert Clegg recommended using an intermediate value of 1.4, which reduces the maximal error in $R_{0}$ to ~ 4% (Clegg, 1992). However, different values may be more appropriate depending on the geometry and environment of the fluorophores. To date, the refractive index has received very little attention in the field (Knox and van Amerongen, 2002).

#### Dye transition dipole orientation factor *κ^2^*

This parameter describes the relative orientation of the transition dipole moments of the dyes and strongly depends on dye mobility. Since the dyes’ orientations can change randomly on the time scale of typical FRET events, the mean value of <*κ^2^>* = 2/3 is typically taken. This well-known dynamic averaging approximation assumes that the rotational diffusion timescale of a FRET pair is much shorter than the fluorescence lifetime of the donor. However, it may well be that one of the dyes is not freely rotating on this timescale (e.g., it may interact with the microenvironment). An extreme example is a FRET system in which non-canonical fluorescent nucleotides were incorporated into dsDNA. The rigid structure and natural helical twist of the DNA caused the relationship between E and $R_{DA}$ to follow an interesting trend (Ranjit et al., 2009) with E being relatively low around $R_{DA}∼R_{0}$, because of *κ^2^* ~ 0 (Wranne et al., 2017). In another smFRET experiment, a DNA molecule was end-labeled with Cy dyes without sulfonic acids groups (Cy3 and Cy5), which have a tendency to stack onto bases at the DNA termini (Iqbal et al., 2008; Ouellet et al., 2011), and the influence of orientational effects on the FRET efficiency was measured. Although an influence of the orientation could be detected, the data showed that orientational effects average-out quite well in most realistic cases (Iqbal et al., 2008). A method to estimate the lower and upper bounds for *<κ^2^>* from the donor and acceptor time-resolved anisotropies was proposed in the 1970s (Dale et al., 1979; van der Meer, 2002). In smFRET measurements using the polarization-resolved MFD modality, information on the donor and acceptor fluorescence intensities, lifetimes, and anisotropies (Schaffer et al., 1999) are collected simultaneously and fluorescence anisotropy decays of different single-molecule sub-populations can be used to assess the *<κ^2^>* uncertainty per conformational state (Ivanov et al., 2009; Kudryavtsev et al., 2012; Sindbert et al., 2011). It is noteworthy to mention that the majority of fluorophores used as donor and acceptor dyes in smFRET have a mono-exponential fluorescence decay and, hence, have one major emission dipole. In this case, the estimation of *<κ^2^>* depends on the orientation of these single transition dipole moments. It has been proposed that the assumption of *<κ^2^> = *2/3 would carry much less uncertainty when the fluorescence signal is emanating from more than one emission dipole, yielding multi-exponential decays (Haas et al., 1978). This is an intriguing idea that could provide a realistic estimation for *κ^2^* and thus help simplify the transformation of FRET efficiencies into inter-dye distances. For a review on the dependence of FRET on *κ^2^*, the reader is referred to (van der Meer, 2002). Finally, we note that there are several routines recommended by experienced members of the community to determine $R_{0}$ accurately. Which approach is the most optimal is still under discussion.

#### Dye models

The Förster equation (Equation 1) allows the extraction of a distance directly from a FRET efficiency measurement. This distance directly corresponds only to the separation of the FRET fluorophores when the positions of the donor and the acceptor molecules are constant, the dye’s orientations are rapidly averaged and their microenvironment is known. Strictly speaking, this is never the case, even for a stable conformation of the labeled macromolecule, since dye molecules are typically attached to the macromolecules via flexible linkers, and the labeled macromolecule usually restricts the volume accessible to the fluorophore (Best et al., 2007; Hellenkamp et al., 2018a; Ingargiola et al., 2018b). In addition, diffusion of the dyes while the donor is in the excited state can also influence the measured FRET efficiency (Ingargiola et al., 2018b). When the FRET rate is not too high (leading to a FRET efficiency of E<0.8) and the dyes do not interact with the protein surface, deviations due to dye dynamics are usually negligible (Hellenkamp et al., 2018a; Hellenkamp et al., 2017; Kalinin et al., 2012).

**Figure 5.**
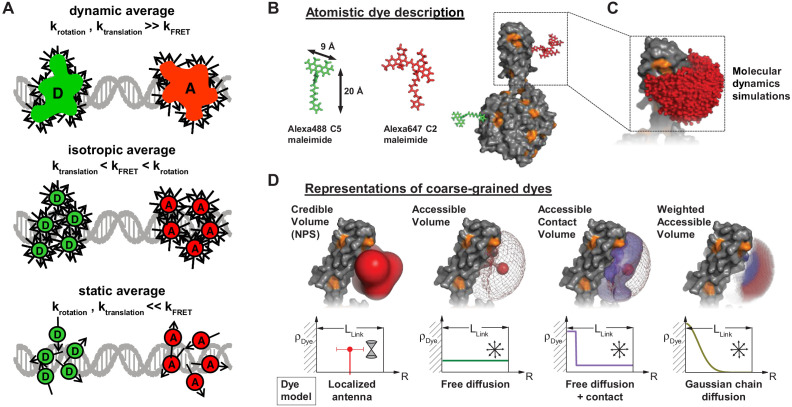
Dye models for FRET. (**A**) The different kinetic averaging regimes for rotation and diffusion are shown schematically. In the dynamic averaging regime, rotation and diffusion happen on a timescale faster than the FRET process. In the isotropic averaging regime, translation is slower than the FRET process, but rotation is fast. The static average applies if both rotation and diffusion are slow compared to the rate of energy transfer. For most experimental situations, the isotropic average is most appropriate. (2008 National Academy of Sciences. Reproduced from Wozniak et al., 2008. Further reproduction of this panel would need permission from the copyright holder.) (**B**) A schematic of a donor/acceptor fluorophore pair (Alexa488, Alexa647) attached to the protein Atlastin-1. (**C**) The accessible volume that the acceptor fluorophore can explore is determined from a molecular dynamics simulation. (**D**) Different coarse-grained dye models are used in the community to describe the three-dimensional dye density ρ_Dye_ (see main text). (Panels B, C, and D were reproduced from Dimura et al., 2016, *Current Opinion in Structural Biology* with permission, published under the Creative Commons Attribution 4.0 International Public License (CC BY 4.0; https://creativecommons.org/licenses/by/4.0/).)

Various groups have developed detailed dye models that account for the translational and rotational flexibility of the dyes and thus allow a more accurate description of the actual distance FRET measures (Figure 5D) (Beckers et al., 2015; Craggs and Kapanidis, 2012; Dimura et al., 2016; Haas et al., 1978; Kalinin et al., 2012; Muschielok et al., 2008; Schuler et al., 2020; Sindbert et al., 2011) and to test them experimentally (Hellenkamp et al., 2018a; Nagy et al., 2018; Peulen et al., 2017; Wozniak et al., 2008). For any given FRET efficiency, different dye models will lead to slightly different extracted $R_{DA}$ distributions (deviations ≤ 5%). Choosing an appropriate model is therefore important for the accurate determination of $R_{DA}$.

Changes in $R_{DA}$ and the relative orientation of the dyes can occur on many different time scales, including the excited state lifetime, the interphoton time, and the photon burst duration. Averaging over these different distances and orientations is complicated due to the inherent non-linearity of the energy transfer process. However, there are exact analyses that describe the photon statistics in smFRET experiments (Antonik et al., 2006; Gopich and Szabo, 2005a; Nir et al., 2006).

Since the dye linker lengths of the typical dyes used in smFRET experiments are long (ranging typically between 10 and 15 atoms, Figure 5B), translational and rotational diffusion of the dyes within the accessible volumes constrained by their linkers and the macromolecules to which they are conjugated lead to considerable changes in $R_{DA}$. Such dynamics can occur on timescales comparable to the fluorescence lifetime, which leads to changes in $R_{DA}$, from the moment of donor excitation to the moment of donor de-excitation. This process leads to the well-documented phenomenon termed diffusion-enhanced FRET (Beechem and Haas, 1989; Haas and Steinberg, 1984; Orevi et al., 2014), where the measured FRET efficiencies are higher than expected from a static distribution of $R_{DA}$ due to the increase in the probability for FRET to occur when $R_{DA}$ shortens while the donor is in the excited state (Eilert et al., 2018; Ingargiola et al., 2018b).

This phenomenon has been treated by incorporating both rotational and translational diffusion of the fluorophores as fluctuations in $R_{DA}$ inside a potential well of the reaction coordinate $R_{DA}$ (Dingfelder et al., 2018; Haas and Steinberg, 1984; Ingargiola et al., 2018b). Similarly, rotational motions lead to changes in the relative orientation of the donor and the acceptor molecules and therefore to changes in $R_{0}$ via *κ^2^*. To this end, a complete kinetic theory treating both rotational and translational diffusion has been developed (Eilert et al., 2018). In many cases, a dynamic rotation - static translation model can be used (i.e., $k_{rotation}>>k_{FRET}>>k_{translation}$) (Figure 5A). Interestingly, Monte-Carlo simulations show that this often-applied simplification can lead to errors in $R_{DA}$ (Hellenkamp et al., 2018a). The magnitude of the uncertainty depends on the donor fluorescence lifetime, the FRET efficiency, and the dye molecules’ diffusion constants and rotational correlation times. So far, no major disagreement of the dynamic rotation – static translation model with experimental data has been reported, thus supporting the use of the isotropic average of <*κ^2^*> = 2/3.

To obtain atomistic insights into the behavior of dyes on biomolecules, molecular dynamics simulations have been explored (Best et al., 2007; Deplazes et al., 2011; Spiegel et al., 2016; Girodat et al., 2020; Grotz et al., 2018; Hoefling et al., 2011; Reinartz et al., 2018; Shoura et al., 2014). By simulating the whole system, including the fluorophores, information is obtained about the accessible volume of the fluorophore, its potential interactions with the biomolecular surface and the dynamics of the system. The results of such simulations crucially depend on the parameterization (force field) of the dyes. Different parameter sets have been reported for commonly used dyes and validated against experimental data (Best et al., 2015; Graen et al., 2014; Schepers and Gohlke, 2020; Shaw et al., 2020), but a consensus on the optimal parameterization has not yet been reached.

### Structural modeling

By accounting for various uncertainties described in the section Inter-dye distances, precise distances can be calculated from FRET efficiencies. This enables the application of FRET for FRET-based structural studies, which are particularly promising for studying the conformations of large, heterogeneous, flexible, and dynamic biomolecules and their complexes (Brunger et al., 2011; Craggs et al., 2019; Filius et al., 2020; Hellenkamp et al., 2017; Holmstrom et al., 2018; Kilic et al., 2018; Muschielok et al., 2008; Nagy et al., 2015; Sanabria et al., 2020; Treutlein et al., 2012; Yanez Orozco et al., 2018). Such systems are notoriously difficult to study with classical structural biology methods. For structure determination, there are further steps that need to be taken.

#### Pipeline

Different approaches have been used to derive structural models from FRET distance restraints. The general pipeline consists of the following steps:

- preparation and measurement of multiple donor-acceptor labeled variants with different labeling positions,
- control experiments to assess the activity after labeling or immobilization, the photophysics of the probes, and the rotational freedom of the dyes,
- the non-trivial transformation from proximity ratios (uncorrected E values) to absolute FRET efficiencies of the different conformational subpopulations, to inter-dye distance information (or equilibrium distance distributions),
- relating the inter-dye distances to the structure by an appropriate dye model, and
- assessing the quality of the structural model.

**Figure 6.**
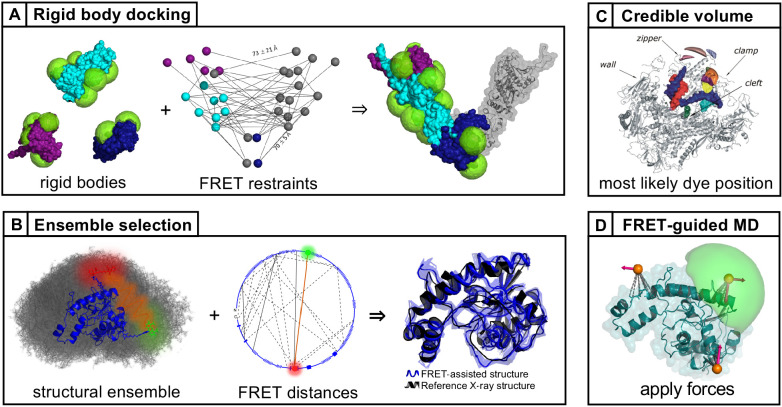
Approaches to integrative modeling using FRET restraints. (**A**) Rigid bodies, representing either different domains of a protein or different proteins within a complex, are arranged by translation and rotation to satisfy the FRET restraints. Adapted from Figure 1 of Hellenkamp et al., 2017. (**B**) The structure that best satisfies all FRET restraints is selected from a prior ensemble. Adapted from Figure 2 of Dimura et al., 2020. (**C**) Using triangulation, the most likely dye position is estimated from the FRET distances with respect to known reference positions on the structure. (Reproduced from Andrecka et al., 2009, Nucleic Acid Research with permission, published under the Creative Commons Attribution-NonCommercial 4.0 International Public License (CC BY NC 4.0; https://creativecommons.org/licenses/by-nc/4.0). Further reproduction of this panel would need permission from the copyright holder.) (**D**) Starting from a known structure, a molecular dynamics simulation is guided using the FRET information by applying forces based on the FRET distances. (Reproduced from Dimura et al., 2020. Further reproduction of this panel would need permission from the copyright holder.)

The FRET information alone is insufficient to generate an atomistic model de novo. FRET-restrained structural modeling thus relies on prior structural knowledge, from which novel structural models are generated. Different approaches have been used, each with specific advantages and limitations (Figure 6).

- Rigid body modeling/docking: The different parts or domains of the structure or complex are treated as rigid bodies and arranged in 3D space to satisfy the FRET restraints (Choi et al., 2010; Hellenkamp et al., 2017; Kalinin et al., 2012; Mekler et al., 2002; Peulen et al., 2020).
- Ensemble selection: An ensemble of structures is generated (e.g., based on MD simulations or normal mode analysis), from which the structures that are best explained by the FRET results are selected (Dimura et al., 2016; Kalinin et al., 2012; Sanabria et al., 2020).
- Credible volumes: The relative position of unresolved structural elements with respect to the known structure is estimated from the FRET-derived distances (Muschielok et al., 2008).
- FRET-guided molecular dynamics: To guide the simulation, FRET-restraints can be incorporated into coarse-grained structural modeling and in all-atom MD simulations (Dimura et al., 2020).

Different laboratories use different approaches to analyze their experimental results in context-dependent manners and the steps necessary for the conversion from FRET information to inter-dye distance information are still under debate. Importantly, the overall uncertainties in inter-dye distances need to be determined from the uncertainty in $R_{0}$, the dye model and experimental precision, and to be incorporated into the integrative structural modeling (Dimura et al., 2016; Hellenkamp et al., 2018a; Hellenkamp et al., 2017; Kalinin et al., 2012; Muschielok et al., 2008; Muschielok and Michaelis, 2011; Peulen et al., 2017). As a result, FRET restraints yielded structural models that showed excellent agreement with existing models in benchmark studies (Kalinin et al., 2012; Mekler et al., 2002), resolved the structure of flexible parts and short-lived excited conformational states that were inaccessible in crystallographic studies (Andrecka et al., 2009; Sanabria et al., 2020) and quantified the conformational flexibility of crystallographic states (Hellenkamp et al., 2017). To increase the success of FRET-derived structural models, a protocol has recently been proposed to determine the most informative labeling positions, the number of labeling positions needed to resolve a given conformation and the accuracy that can be expected for the FRET-derived structural models (Dimura et al., 2020).

We should also be aware of the expected uncertainty for the determined structural model, which can be computed from the number of measurements, their average quality and the properties of the underlying computational method. Besides estimating the precision (Dimura et al., 2020), it is useful to introduce a quantitative quality estimate, $\chi_{n}^{2}$, for judging the accuracy of FRET-derived structure models based on a cross-validation statistical method in the spirit of $R_{free}$ used in X-ray crystallography (Brünger, 1992). Such accuracy estimates are essential for the quality control of I/H structure models, especially those deposited in the PDB-Dev. Dimura et al., 2020 pointed out that the complexity arising from different experiments could be mitigated by using Bayesian hierarchical data processing frameworks, which abstracts the experimental data and propagates the information and uncertainties to enable structural modeling at higher precision and accuracy. Combining the smFRET information with additional constraints provided by complementary methods has the potential to further improve the accuracy of the obtained I/H structural models (Berman et al., 2019).

### Verification of steps in the workflow using simulations

At various points along the workflow, simulations can be extremely useful. They can be used to gain a better understanding of the analysis procedures (e.g., to test the impact of specific burst search and selection parameters) (Hagai and Lerner, 2019) or to determine to what extent a particular data analysis procedure is capable of extracting results reliably (e.g., distinguishing between one or two subpopulations) (Blanco and Walter, 2010; Chen et al., 2016). Having a ‘ground truth’ to compare to is also very helpful when developing new analysis methods before applying it to real data. This question is relevant, especially when other common analysis procedures yield different results. Of course, this practice should not replace control measurements and analyses with an established system for validation of a given method, although numerical simulations can give an economical and initial guidance. Using prior knowledge about the measured system and the experimental setup, one can perform simulations mimicking real measurements and then check the results of different analysis procedures. In this way, it is possible to determine which analysis procedure is most promising in a given context. Another insightful aspect that simulations can provide is a consistency check of the data. One can simulate smFRET data assuming the number of states and/or kinetic rates derived from the experiments to see how well the model describes the measured data.

## Open science

One of the cornerstones of the scientific method is the ability to reproduce experimental results. As experiments become more sophisticated, a clear description of the experiments is crucial. Recent trends toward Open Science practices call for full transparency of the scientific process, as has been formulated in the FAIR principle that data should be ‘Findable, Accessible, Interoperable, and Re-usable’ (Wilkinson et al., 2016). To this end, the procedures taken to acquire, analyze, and interpret experimental data should be provided. That includes describing each step, the reasons for taking the step and the information associated with the step. To ensure that the analysis remains transparent and tractable, code should generally be openly available and all parameters and settings used in the analysis should be stored.

Funding agencies embracing this philosophy (e.g., https://datascience.nih.gov/strategicplan) expect grantees to publish in open-access (OA) journals (and pay for the corresponding OA fees) or deposit manuscripts on preprint servers (e.g., Pubmed Central, arXiv, bioRxiv, ChemRxiv, medRxiv), and deposit data (sometimes also raw data) in repositories (e.g., Zenodo, the Dryad Digital Repository, FigShare) as well as analyses codes (e.g., GitHub). Open science disseminates knowledge by freely sharing results and the tools developed by independent scientists or teams working as part of a collaborative network. We would like to see the FRET community embrace and be committed to open science. Some tools are already in place, while others still need to be developed to make it easier to communicate the continuously growing knowledge and experience present in the FRET community.

### Intellectual property and software licenses

There is obviously some tension between the precepts of open science and requirements imposed by some intellectual property (IP) policies. IP rights, including patent laws, were put in place to promote the development of science and technology for the benefit of society by allowing those developing intellectual property to retain the rights for the IP they developed. In fact, in some sense, patents were the first form of open access publication, only with a restrictive license for reuse. We do not oppose intellectual property rights, but given the developmental stage the FRET field is currently in, we support the disclosure of methods, data, and software. For the advancement of the field, other groups must be able to reproduce the analyses of existing data, extend upon them and, if needed, be able to reproduce the experiments. The acquisition and analysis must be modifiable and extendable in agreement with the license chosen by the data or software creator. This license should be set as liberal as possible, taking into account the IP considerations mentioned above, but also encourage recognition of the considerable effort invested in producing successful protocols, designs, data, or software. Ultimately, if practiced fairly, open science should entice everyone, including commercial vendors, to adopt and contribute to community-defined file formats, provide free file-conversion codes, and open their analysis tools for scrutiny by the community.

### Proper documentation of data analysis practices

By making analysis codes and protocols freely available, we hope to stimulate the acceptance, utilization, and exchange of new methods and tools. It is true that there already exist a large number of open-access programs that offer a large variety of analysis procedures for single-molecule photon trajectories (free-diffusion smFRET) and single-molecule videos (immobilized smFRET) data (see Table 1).

**Table 1.** List of available software for smFRET analysis.

Software	Type	Description	URL
ALiX	Confocal	ALiX is developed for basic research on diffusing two-color smFRET in single or multiple spot geometries (Ingargiola et al., 2017).	https://sites.google.com/a/g.ucla.edu/alix/
Fretica	Confocal	Fretica, a Mathematica package with a backend written in C++, is a user-extendable toolbox that supports MFD, PIE/ALEX, PCH (Huang et al., 2004; Müller et al., 2000), FIDA (Gopich and Szabo, 2005b; Kask et al., 1999), PDA (Antonik et al., 2006; Ernst et al., 2020), recurrence analysis (Hoffmann et al., 2011), fluorescence lifetime fitting, FLIM, FCS, FLCS (Arbour and Enderlein, 2010; Dertinger et al., 2007), dual-focus FCS, nsFCS (Nettels et al., 2007; Schuler and Hofmann, 2013), maximum likelihood estimation from photon-by-photon (Gopich and Szabo, 2009) and binned trajectories, simulation of confocal experiments and more (Nettels and Schuler, 2020; https://schuler.bioc.uzh.ch/programs/).	https://schuler.bioc.uzh.ch/programs/
FRET_3colorCW	Confocal	C++ and MATLAB GUI-based CPU-GPU co-parallelization software package for an enhanced maximum likelihood analysis of two- and three-color fluorescence photon trajectories generated by continuous-wave donor excitation (Yoo et al., 2020).	https://github.com/hoisunglab/FRET_3colorCW
gSMFRETda	Confocal	gSMFRETda is a GPU-capable program for Monte Carlo simulations of PDA. It can sample dwell time and other parameters in fine grids, thus allowing the analysis of rapid dynamic interconversions (Liu et al., 2019).	https://github.com/liu-kan/gSMFRETda
H^2^MM	Confocal	H^2^MM is a maximum likelihood estimation algorithm for a photon-by-photon analysis of smFRET experiments (Pirchi et al., 2016).	http://pubs.acs.org/doi/suppl/10.1021/acs.jpcb.6b10726/suppl_file/jp6b10726_si_002.zip
MFD Spectroscopy and Imaging	Confocal	A modular software package for confocal fluorescence spectroscopy and imaging experiments using multiparameter fluorescence detection (MFD) with all tools (FCS, fFCS, PDA, seTCSPC, trace analysis, 2D simulation of MFD diagrams, Burbulator) and multiparameter fluorescence image spectroscopy (MFIS) (Antonik et al., 2006; Felekyan et al., 2005; Kühnemuth and Seidel, 2001; Widengren et al., 2006; Felekyan et al., 2020).	https://www.mpc.hhu.de/software/3-software-package-for-mfd-fcs-and-mfis
OpenSMFS	Confocal	A collection of tools (Ingargiola et al., 2016b) for solution-based single-molecule fluorescence spectroscopy, including smFRET, FCS, MC-DEPI (Ingargiola et al., 2018b).	https://github.com/OpenSMFS
smfBox	Confocal	A confocal smFRET platform, providing build instructions and open-source acquisition software (Ambrose et al., 2020).	https://craggslab.github.io/smfBox/
rFRET	Confocal Imaging	rFRET is a comprehensive, MATLAB-based program for analyzing ratiometric microscopic FRET experiments (Nagy et al., 2016).	https://peternagy.webs.com/fret#rfret
ChiSurf	Confocal Imaging Ensemble	ChiSurf is a fluorescence analysis platform for the analysis of time-resolved fluorescence decays (Peulen et al., 2017).	https://github.com/Fluorescence-Tools/ChiSurf/wiki
PAM - PIE Analysis with MATLAB	Confocal Imaging Ensemble	PAM (PIE analysis with MATLAB) is a GUI-based software package for the analysis of fluorescence experiments and supports a large number of analysis methods ranging from single-molecule methods to imaging (Schrimpf et al., 2018; Barth et al., 2020).	RRID:SCR_020966, https://www.cup.uni-muenchen.de/pc/lamb/software/pam.html
AutoSiM	TIRF	AutoSiM is a deep-learning developed MATLAB program for automatically selecting and sorting smFRET traces (Li et al., 2020a).	https://doi.org/10.7302/ck2m-qf69
BIASD	TIRF	BIASD uses Bayesian inference to infer transition rates that are more than three orders of magnitude larger than the acquisition rate of the experimental smFRET data (Kinz-Thompson and Gonzalez, 2018).	http://github.com/ckinzthompson/biasd
DeepFRET	TIRF	smFRET software based on deep-learning for automatic trace selection and classification. It includes all common features: image analysis, background-corrected trace-extraction, hidden Markov analysis, correction factor application, and data visualization (Thomsen et al., 2020).	https://github.com/hatzakislab/DeepFRET-GUI
ebFRET	TIRF	ebFRET performs combined analysis on multiple smFRET time-series to learn a set of rates and states (van de Meent et al., 2014).	https://ebfret.github.io/
FRETboard	TIRF	smFRET data preprocessing and analysis using algorithms of choice and user supervision. Also offered as a web-based user interface (de Lannoy et al., 2020).	https://github.com/cvdelannoy/FRETboard
HaMMy	TIRF	smFRET analysis and hidden Markov modeling (McKinney et al., 2006).	http://ha.med.jhmi.edu/resources/
hFRET	TIRF	hFRET uses variational Bayesian inference to estimate the parameters of a hierarchical hidden Markov model, thereby enabling robust identification and characterization of kinetic heterogeneity (Hon and Gonzalez, 2019).	https://github.com/GonzalezBiophysicsLab/hFRET
iSMS	TIRF	iSMS is a user-interfaced software package for smFRET data analysis. It includes extraction of time-traces from movies, traces grouping/selection tools according to defined criteria, application of corrections, data visualization and analysis with hidden Markov modeling, and import/export possibilities in different formats for data-sharing (Preus et al., 2015; Preuss et al., 2020).	http://isms.au.dk/
MASH-FRET	TIRF	MASH-FRET is a MATLAB-based software package for the simulation (Börner et al., 2018) and analysis of single-molecule FRET videos and trajectories (video processing [Hadzic et al., 2016], histogram analysis [König et al., 2013], and transitions analysis [Hadzic et al., 2018; König et al., 2013]).	https://rna-fretools.github.io/MASH-FRET/
miCUBE	TIRF	TIRF smFRET platform, providing detailed build instructions and open-source acquisition software (Martens et al., 2019).	https://hohlbeinlab.github.io/miCube/index.html
SMACKS	TIRF	SMACKS (single-molecule analysis of complex kinetic sequences) is a maximum-likelihood approach to extract kinetic rate models from noisy single-molecule data (Schmid et al., 2016).	https://www.singlemolecule.uni-freiburg.de/software/smacks
smCamera	TIRF	smFRET data acquisition (Windows. exe) and analysis (IDL, MATLAB) with example data (Roy et al., 2008).	http://ha.med.jhmi.edu/resources/
SPARTAN	TIRF	Automated analysis of smFRET multiple single-molecule recordings. It includes extraction of traces from movies, trace selection according to defined criteria, application of corrections, hidden Markov modeling, simulations, and data visualization (Juette et al., 2016).	https://www.scottcblanchardlab.com/software
STaSI	TIRF	STaSI uses the Student’s t-test and groups the segments into states by hierarchical clustering (Shuang et al., 2014).	https://github.com/LandesLab/STaSI
TwoTone	TIRF	A TIRF-FRET analysis package for the automatic analysis of single-molecule FRET movies (Holden et al., 2011).	https://groups.physics.ox.ac.uk/genemachines/group/Main.Software.html
vbFRET	TIRF	vbFRET uses variational Bayesian inference to learn hidden Markov models from individual, smFRET time trajectories (Bronson et al., 2009).	http://www.columbia.edu/cu/chemistry/groups/gonzalez/software.html
Fast NPS	Modeling	A nano-positioning system for macromolecular structural analysis (Eilert et al., 2017).	http://dx.doi.org/10.17632/7ztzj63r68.1
Fluordynamics	Modeling	Fluordynamics is a PyMOL plugin to label biomolecules with organic fluorophores for all-atom molecular dynamics simulations. It builds on AMBERDYES (Schepers and Gohlke, 2020) and extends the force field to common nucleic acid linker chemistries (Steffen et al., 2016).	https://github.com/RNA-FRETools/fluordynamics
FPS	Modeling	A toolkit for FRET restrained modeling of biomolecules and their complexes for quantitative applications in structural biology (Kalinin et al., 2012).	https://www.mpc.hhu.de/software/1-fret-positioning-and-screening-fps
FRETraj	Modeling	FRETraj is a Python API to the LabelLib package, which integrates into PyMOL to interactively calculate accessible-contact volumes and predict FRET efficiencies (Steffen et al., 2016).	https://github.com/RNA-FRETools/fretraj
FRETrest in Amber20	Modeling	FRETrest is a set of helper scripts for generating FRET-restraints for Molecular Dynamics (MD) simulations performed with the AMBER Software Suite (Dimura et al., 2020).	http://ambermd.org/doc12/Amber20.pdf
LabelLib	Modeling	LabelLib is a C++ library for the simulation of the accessible volume (AV) of small probes flexibly coupled to biomolecules (Dimura et al., 2016; Kalinin et al., 2012).	https://github.com/Fluorescence-Tools/LabelLib

To further improve the inter-operation between methods and analysis and to establish convenient documentation protocols, it is essential to work in an open multivalent environment. For this goal, the use of browser-based software such as ‘*Jupyter* notebooks’ and/or other available workspaces may serve as a convenient platform. Such workspaces provide an interactive scripting environment by combining formatted ‘rich’ text with well-commented code commands as well as code outputs (e.g., figures, tables, comments, equations) and explanations in a single web-based document. Such web-based workspace environments support several programming languages, including Python, R, C++, and, to some extent, MATLAB and Mathematica. Practitioners using this environment can then easily read, distribute, re-run, check, and modify the code. Software engineering approaches in scientific software usually include version control, code review, unit testing, continuous integration, and auto-generation of HTML manuals. In the next step, *Jupyter* notebooks or similar workspaces can also help newcomers perform complex analyses already in the web-based environment with minimal adaptation efforts, which will accelerate the dissemination of new analyses. Indeed, well-documented, easy-to-use notebooks have been provided by various groups (Ambrose et al., 2020; Ingargiola et al., 2016b; Ingargiola et al., 2016a; Lerner, 2020; Lerner, 2019) (e.g., at https://github.com/tritemio/FRETBursts or https://craggslab.github.io/smfBox/).

Although the notebook approach offers advantages to experienced users and software developers, it might be difficult for many end-users to adapt to the script-based workflow. For those users, it may be more convenient to use the established and tested algorithms embedded in a graphical user interface (GUI). Indeed, there is a large variety of user-friendly software available (compiled in Table 1). To further increase the ease of use, the *FRETboard* software aims to make the underlying analysis algorithms of other packages available through a single web-based GUI (de Lannoy et al., 2020). As it can be used in a browser through a remote web server, this would allow any user to freely experiment with different analysis methods without the need for software installation or heavy computational resources.

As the first step toward FAIR-compliant analysis practices, we propose establishing a software library that contains tested and proven algorithms for the analysis of fluorescence experiments, which will assist in their efficient distribution and implementation in existing workflows. Such efforts have already been initiated in the *FRETbursts* software package (Ingargiola et al., 2016b), and a GitHub group has been established at https://github.com/Fluorescence-Tools to collect software packages and connect software developers. Establishing a community-wide working group of ‘*Analysis software for FRET*’ would be an important step in organizing and moderating this process.

### Standard file format

To expedite the exchange of data between different groups and testing of different analysis methods, it would be beneficial to have a minimal number of file formats, and to avoid the multiplication of ad hoc formats developed independently. In fact, the absence of a standard file format and supporting documents has caused issues within individual labs with respect to long-term data storage and re-analysis. Online data deposition in well-documented file formats would therefore save a lot of headaches for many laboratories. Even if standard file formats are carefully designed or developed, it will be inevitable to modify the existing formats or introduce new formats in the future. The list of standard formats should be regularly updated, and the analysis codes should also be kept current and properly maintained to guarantee their *compatibility* with the new standard formats.

**Figure 7.**
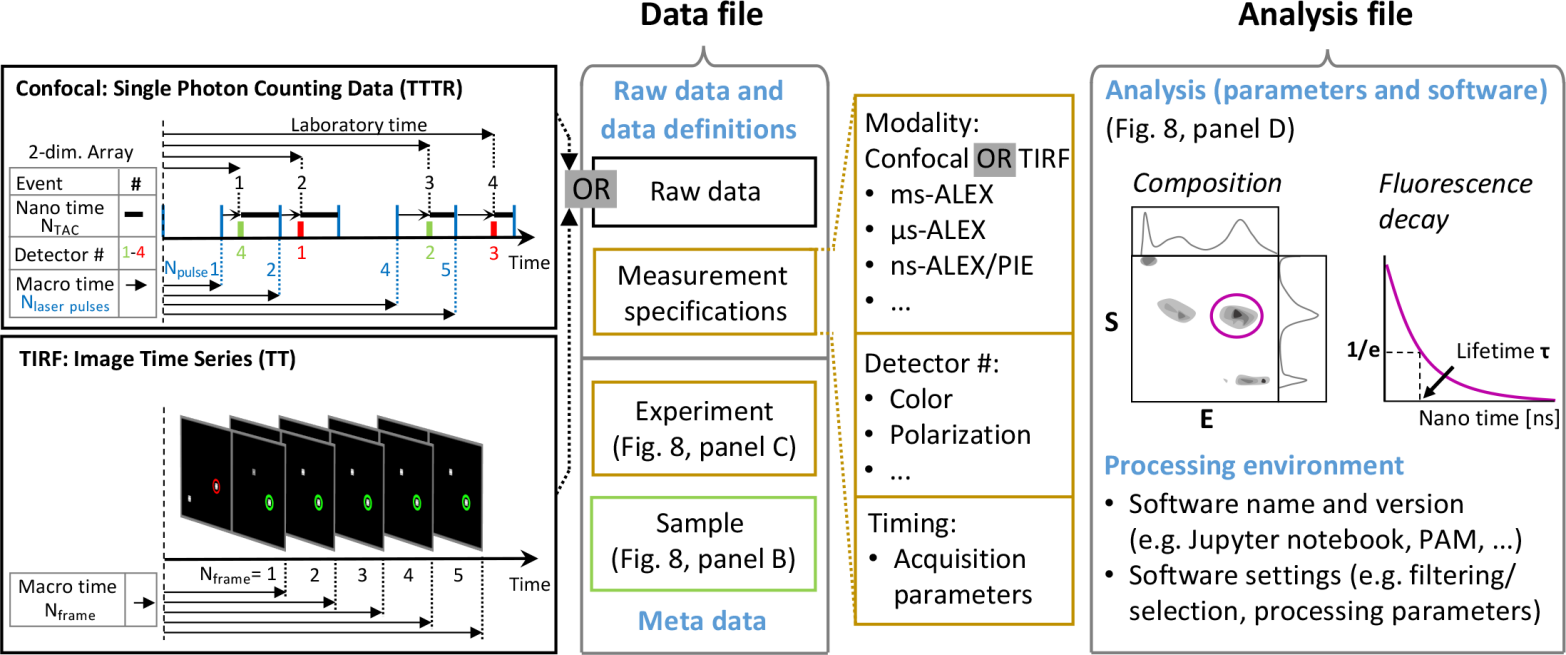
Concept for data storage following the FAIR principle. All essential information should be contained in two data files, one for raw data and a second with the essential information regarding the associated analysis. (*Left*) Structure of proposed data file formats containing confocal or TIRF raw data in a time-tagged, TT, or time-tagged time-resolved, TTTR, format together with sample- and experiment-specific metadata (for details, see Figure 8). The measurement specifications are needed by the reading routine to reconstruct the photon trace from the stored data, for example, for timing in the TT format (sampling frequencies expressed as time bins or frame rates) or in the TTTR format (laser repetition rates, time binning in time-correlated single-photon counting). Moreover, the detector that measured the signal is noted (detector #) along with the detection time with a given time resolution. For representing the detection time of single photons in time-resolved smFRET studies, the TTTR format is used where the time corresponds to the sum of the macro time and the nano time (upper left panel). The macro time comprises multiple cycles of excitation laser pulses (blue vertical lines) and the nano time is determined by time-correlated single-photon counting with picosecond resolution. The TTTR format is the most compact data format for single-molecule fluorescence data for detection times with picosecond time resolution and macro-times of hours (Felekyan et al., 2005). The representation of the photon detection of intensity-based and imaging-based smFRET studies is in the TT format, where the macro-time comprises multiples of the external clock pulse or readout cycles, where single or several photons were detected. The stack of TIFF images acquired in TIRF measurements (lower left panel) is transformed into the TT format for analyzing photon time traces for selected spots. (*Middle*) For the corresponding data file, a metadata system as implemented in the Photon-HDF5 file format (Ingargiola et al., 2016a) is suggested. (*Right*) The analysis file should contain the determined parameters obtained by a quantitative analysis together with analysis metadata that assure evaluation, reproducibility, and re-usability of the analysis. The processed data should be documented as outlined in Figure 8D.

The raw experimental data should be supplied in a universal data file format that can be easily read and scrutinized (Figure 7 left). Ideally, the file should store both raw data and sufficient metadata to specify the measurement, setup, and sample. The metadata should be stored in a human-readable text-based format, while space-efficient storage of the raw photon data should be ensured by lossless compression. There are currently many different file formats for smFRET data, developed by different research groups and companies. In general, such files are hard to access for other laboratories and they are not guaranteed to be perennial, which poses an additional challenge to the community. To promote the adaptation of new file formats, conversion tools for older file formats should be provided so that future software codes can focus on handling one (or at least only a very few) common file formats, such as the *phconvert* suite of notebooks for transforming many file formats into Photon-HDF5 (see http://photon-hdf5.github.io/phconvert/). Reaching such a consensus is possible, as has been demonstrated by the acceptance of a single file format for the deposition of NMR data (Ulrich et al., 2019).

#### File formats for (time-correlated) photon counting with point-detector data (confocal modality)

Several formats have been used and reported for time-correlated single-photon counting data (Brand et al., 1997; Brooks Shera et al., 1990; Eggeling et al., 2001; Felekyan et al., 2005; Rigler et al., 1993; Schaffer et al., 1999; Tellinghuisen et al., 1994; Wahl et al., 2008; Widengren et al., 2006). One example is the time-tagged time-resolved (TTTR) file format given in the left panel of Figure 7. Because of its compactness, this file format has been widely adopted by commercial companies (e.g., Becker and Hickl and PicoQuant) providing TCSPC electronics and point detectors for solution/imaging studies.

The basic formats have been extended in the Photon-HDF5 (Ingargiola et al., 2016a) file format that connects rich metadata with the raw photon information in a single, space-efficient format suitable for sharing and long-term data archival. Moreover, several software programs exist that can easily transform raw data files to the Photon-HDF5 format. Once enough metadata is available in the community, the relative importance of particular metadata entries on the resulting FRET data can be assessed.

#### File format for camera-based acquisition (wide-field/TIRF modality)

Camera-based data is acquired as a stack of images (e.g., TIFF). To extract time trajectories, several steps are needed to yield the time-binned fluorescence intensity (e.g., spot identification; donor and acceptor spot registration; thresholding; background subtraction, etc.). A file format for TIRF-based smFRET (immobilized) measurements has been proposed (Greenfeld et al., 2015). Alternatively, human-readable plain text files with an agreed format and greatly downsized from the raw TIFF stacks can also work well for TIRF-based smFRET traces. Binary file formats for efficient data compression have also been implemented (Juette et al., 2016).

#### Exchange file format for processed data

In addition to standardized raw file formats, we recommend defining exchange file formats for different levels of processed data (Figure 7, middle panel). This will allow researchers to establish flexible and modular workflows spanning different software packages and facilitate the adoption of novel analysis approaches. The deposition of processed data in agreed-upon file formats also ensures that published data can be re-used at a later time point, for example, for more elaborate structural modeling approaches (Köfinger et al., 2019). For the storage of processed data, we believe that it is important to retain the connection to the raw data by including the relevant metadata. For example, corrected FRET efficiency histograms should be deposited together with the raw signal intensities in the donor and acceptor channels, the background intensities and the calibration factors.

### Repositories and data bank for FRET data and models

#### Metadata for FRET experiments

**Figure 8.**
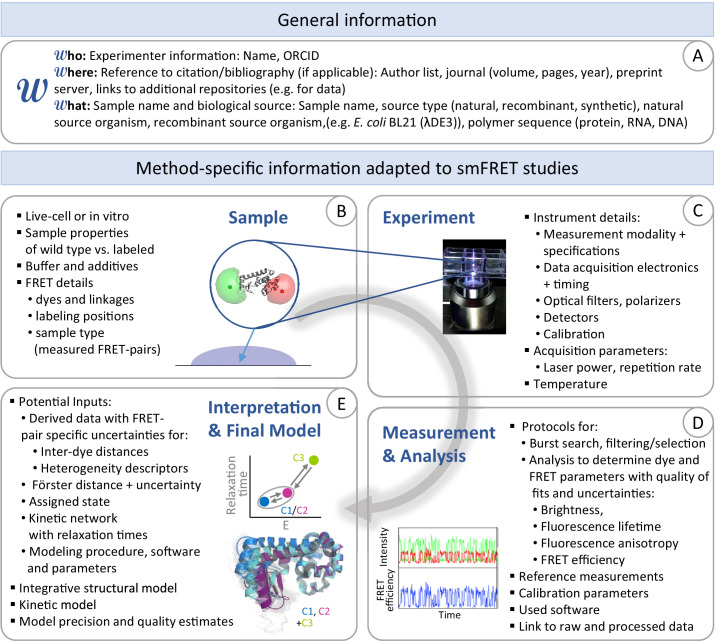
Disseminating the results of smFRET studies. Recommended categories for data and method-specific information (metadata), which are needed for documentation of smFRET studies where the authors want to archive their obtained kinetic/structural models. (**A**) General information. (**B**) Information on the sample and FRET-specific properties. (**C**) Information on the experiment and data acquisition. (**D**) Information on processing and analysis procedures. (**E**) Information on the interpretation of the data and the final kinetic network or structural model.

To ensure that the reported results are reproducible, raw data must be sufficiently annotated. One important source of inspiration for developing recommendations and standards for the FRET community comes from the world-wide Protein Data Bank (wwPDB) (Berman et al., 2003; Young and Westbrook, 2019). Following the standards of the wwPDB, it is recommended to provide additional information to the models as outlined in Figure 8.

#### Archiving FRET experiments

In this respect, we strongly encourage the publication of datasets with detailed descriptions of the acquisition and analysis in scientific journals. Alternatively, or in addition, structural information and raw datasets should immediately be deposited in repositories provided by the publisher or generalist repositories (Table 2) with a Digital Object Identifier or link for access and citation. Online and public repositories also act as a form of data and knowledge backup, which is difficult to achieve and maintain at the scale of a single laboratory. Comparing a repository with a database, it is obvious that a database has significant advantages for the scientific community. While both archive forms provide open access to the data for all users, only a database can fulfill the quality criteria for safe usage of the deposited data by (1) creating standard definitions for the experimental data used for determining kinetic and/or structural models and their features; (2) developing methods to collect the minimum amount of required information for curation and validation of models and data; and (3) building the infrastructure for acquiring, archiving and disseminating the models and the data. To conclude, a database assures appropriate documentation with a defined format and quality control of the results.

**Table 2.** A list of repositories for citable data storage.

Repository name	URL	Size limits	Fee/Costs
Dryad Digital Repository	https://datadryad.org/stash	No	$120 USD for first 20 GB, and $50 USD for each additional 10 GB
figshare	https://figshare.com/	1 TB per dataset	It varies with publishers
Harvard Dataverse	https://dataverse.harvard.edu/	2.5 GB per file, 10 GB per dataset	Free
Open Science Framework	https://osf.io/	5 GB per file, multiple files can be uploaded	Free
Zenodo	https://zenodo.org/	50 GB per dataset	Donation
Mendeley Data	https://data.mendeley.com/	10 GB per dataset	Free

Given the importance of integrative structures for advancing life sciences and the significant world-wide investment made to determine them, the wwPDB has proposed a governance structure for federating archives containing structural models (e.g., PDB, Electron Microscopy Data Bank, EMDB Tagari et al., 2002) or experimental data. Moreover, for integrative modeling integrative/hybrid modeling structures (Sali et al., 2015), PDB-Dev (Berman et al., 2019; Vallat et al., 2018) was established as an associated prototype system for archiving multi-state and ensemble structures on multiple spatial scales. In addition, associated kinetic models can be deposited.

To deposit structural models and kinetic networks obtained using FRET experiments in PDB-Dev, a dictionary for FRET data is under development (https://github.com/ihmwg/FLR-dictionary; Vallat et al., 2020), serving as a method-specific extension to the existing integrative/hybrid modeling (IHM) dictionary that contains the data categories described in Figure 8. Presently, three integrative FRET-assisted structural models can be found on PDB-Dev.

In addition to archiving the models themselves, all relevant experimental data and metadata should be archived in a technique-associated data bank similar to the Biological Magnetic Resonance Data Bank, BMRB (Ulrich et al., 2019; Ulrich et al., 2008). Reaching such a consensus is possible, as has been demonstrated by the acceptance of a single file format for the deposition of NMR data (Ulrich et al., 2019). In the future, federated resources for other biophysical techniques are expected to align with the structural model archives of the wwPDB for participating in data exchange. Thus, it would be desirable to establish a federated data bank for archiving FRET and, more generally, fluorescence data that could be referred to as the Fluorescence Data Bank (FLDB).

## Community actions to bring FRET scientists together

To better achieve a consensus on the current and the future directions of the smFRET community, an open forum is needed where the current issues, needs, and desires could be discussed. We propose the following tools to organize the community around standardization efforts and open science practices. Some of these tools have already been put in place.

### Community website as a central hub

A website for the FRET community has been established at https://www.fret.community. The community is open to everybody and registered members can populate their user profiles with additional information such as a description of their scientific interests or a list of key-publications. Besides providing regular updates on the activities within the community, the website also provides resources such as a curated list of software packages (see Table 1) and offers a discussion platform through an integrated forum. The website serves as a platform for ongoing discussions, announcements of accepted relevant papers, notifications about upcoming meetings, workshops, competitions and other activities that might be relevant to the community. An advisory board, elected by the community, moderates the website. One can also envision adding an educational section, much like the popular website for general microscopy education (https://micro.magnet.fsu.edu).

### Listserv

To facilitate the dissemination of important information to the FRET community, an electronic mailing list (Listserv) has been established. In order to subscribe to it, smFRET practitioners are requested to register (free of charge) using the following link: https://www.fret.community/register. The members will be informed through the email list about ongoing activities and developments within the community, such as experimental or computational challenges, key publications in the fields, and workshops or meetings.

### Server and repository

A repository will be established, which will be accessible through the community website, to host a collection of software packages and facilitate the community-driven joint development of analysis tools. The repository will contain dedicated sections for acquisition software, raw data, analysis codes, analyzed data files, and file conversion utilities. In order to deposit code in the repository, guidelines for the required documentation will be provided.

The concept of the repository is to support open science and transparency. Anyone registered on the website will be able to access raw data, and analyze and compare performances of the various analysis codes. Moreover, the codes can be updated and expanded (while keeping original versions) by anyone. In this way, improvements and enhancements can be implemented and tested. In that context, it is important to mention that such a repository can also serve the purpose of source data deposition, nowadays required by many scientific journals.

### Participation in CASP(-like) competitions

Critical Assessment of protein Structure Prediction (CASP, http://predictioncenter.org/) is a grass-roots effort for predicting a three-dimensional protein structure from its amino acid sequence. CASP has been run, since 1994, as a double-blind competition. It provides research groups with an opportunity to test their structure prediction methods objectively. CASP has been exploring modeling methods based, in part, on sparse experimental data, including data from SAXS, NMR, crosslinking, and FRET. This integrative CASP experiment was highlighted at the recent CASP13 meeting (http://www.predictioncenter.org/), where the carbohydrate-binding module (CBM56) of a β−1,3-glucanase from *Bacillus circulans* with 184 amino acids (18.9 kDa) was studied as the first FRET data-assisted target F0964. In CASP14, the single-model protein structure prediction by the artificial intelligence (AI) network AlphaFold2, which was developed by Google's AI offshoot DeepMind (https://deepmind.com), has approached perfection (Callaway, 2020). This deep-learning program combines the evolutionary information from multiple sequence alignments with structural information from the PDB for computing 3D structural models of a protein from its amino-acid sequence.

However, one has to be aware that many proteins do not only adopt their thermodynamically most stable conformation but frequently exist as ensembles of conformations that have high functional relevance. Thus, mapping dynamic ensembles represents the subsequent challenge of structural biology for the next decades. Due to their high time-resolution, smFRET-studies and integrative modeling can contribute a lot to solving this problem. We propose that members of the smFRET community who are interested in using smFRET for integrative structural biology participate in the CASP competition. Involvement could progress in several stages: (1) Predicting single- and multi-state structural models: the smFRET community will only submit distances that will be evaluated with respect to the known (but undisclosed) crystal structure(s). (2) Predicting ensembles as in the case of CBM56: for targets that are identified as difficult by the predictors and for which multiple possible folds are submitted without a clear winner, a FRET-assisted round could be insightful where the FRET distances distributions can be used as an experimental ‘ground truth’ for checking whether multiple conformations in an exchange are present.

These recommendations apply mostly to present and future practitioners of smFRET-driven integrative modeling. That being said, smFRET is one of many biophysical techniques that can provide experimental restraints in integrative modeling (XL-MS, single-particle cryoEM, NMR, SAXS). Therefore, we propose that, at a later stage, an all-biophysics integrative structural biology competition be established.

### SmFRET meetings

Several gatherings of FRET practitioners at the Annual Biophysical Society Meetings, supported by the Biological Fluorescence subgroup, provided a platform for planning future activities and establishing the FRET community. As further joint actions, satellite meetings to the Conference on Methods and Applications in Fluorescence (MAF) have been organized to discuss practices, standards, competitions, and joint publications. We envision an occasional dedicated meeting for the smFRET community, such as the Bunsen meetings on FRET held in 2011 and the international discussion meeting in 2016 at the Max Planck Institute for Biophysical Chemistry in Göttingen, Germany (http://fret.uni-duesseldorf.de/cms/home.html). However, to open these meetings to smFRET practitioners outside of Europe, we propose to rotate the venue among continents. We also suggest using the satellite meetings and workshops to disseminate information (details of accurate FRET measurements, common practices, standards and competitions) and to give newcomers the chance to interact with the experienced researchers in the field.

Inspired by the online seminars emerging in response to the COVID-19 pandemic, smFRET webinars and web conferences open to all should be pursued. They provide FRET researchers the unique opportunity to attend and socialize virtually and would be a forum for good scientific practice of open science for the FRET community.

### Special issues in journals

To further stimulate newcomers to engage in advanced smFRET experiments, the FRET community could benefit by hosting special issues in journals dedicated to data analyses (e.g., *Data in Brief, Methods in Molecular Biology, or Nature Protocols*). Here, various laboratories can describe typical datasets or protocols for the methods they have developed. Also, journals disseminating methodologies and protocols from A-to-Z via video recordings could be useful. For example, there is a special issue focusing on FRET planned in the *Journal of Visualized Experiments* (https://www.jove.com/methods-collections/682).

## Future of smFRET

With improved communication and dissemination within the FRET community and agreement on the standard information required for depositing FRET-based or integrative structural data, smFRET will be better positioned to impact the expanding field of dynamic structural biology. We expect integrated approaches such as combining smFRET with NMR (Aznauryan et al., 2016; Liu et al., 2018; Milles et al., 2015; Sottini et al., 2020; Tsytlonok et al., 2019; Voith von Voithenberg et al., 2016), EPR (Boura et al., 2011; Masliah et al., 2018; Peter et al., 2020; Peulen et al., 2020; Sanabria et al., 2020; Vöpel et al., 2014), cross-linking mass spectrometry (Calabrese et al., 2020; Liu et al., 2018; Tyagi and Lemke, 2015), hydrogen/deuterium exchange (Calabrese et al., 2020; Liu et al., 2018; Munro and Lee, 2018), and/or MD simulations (see section Structural modeling and below) will have a big impact in the future.

**Figure 9.**
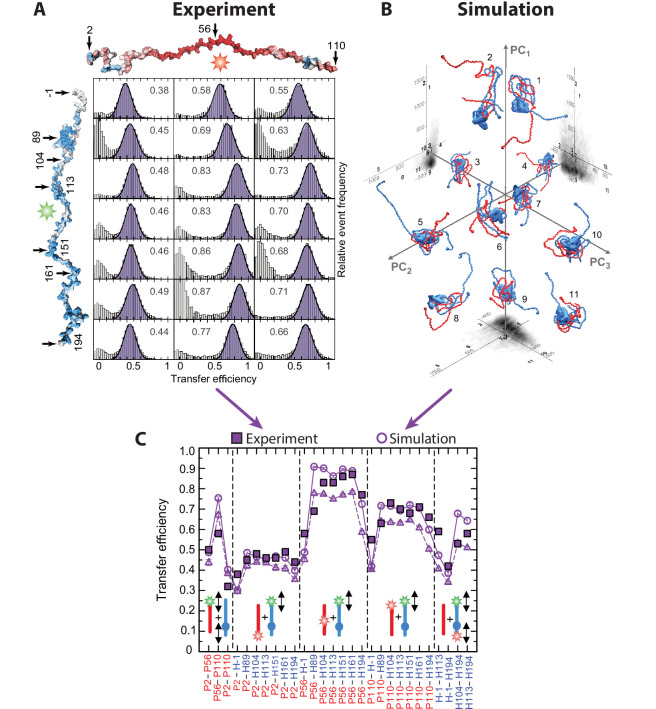
Using smFRET to investigate the structure and dynamics of ultrahigh-affinity IDP complexes. (**A**) SmFRET efficiency histograms for FRET between a donor label (Alexa488) attached at various positions to the linker histone H1 (shown in blue) with the IDP ProTα (shown in red) labeled at different positions with the acceptor fluorophore (Alexa594). (**B**) For structural calculations of the H1-ProTα complex, coarse-grained MD simulations were performed. From the MD simulations, an ensemble of structures was determined. Eleven examples of configurations are shown and projected onto the first three principle components (PC1, PC2, and PC3) of the inter-residue distance map. 2D projections of the full ensemble are shown in gray (axes are labeled in Å). (**C**) A comparison of the experimental FRET efficiencies (filled squares) and the FRET efficiencies estimated from simulated structures (open circles) shows good agreement between the measured and simulated values. Pictograms indicate the variations of dye positions studied. (Panels **A**, **B**, and **C**: Copyright 2018, Nature Publishing Group, a division of Macmillan Publishers Limited. All rights reserved. Reproduced from Borgia et al., 2018, with permission. Further reproduction of this panel would need permission from the copyright holder.)

One example of a major area of interest that is profiting from these developments is the study of intrinsically disordered proteins using smFRET experiments (Gomes and Gradinaru, 2017; Gomes et al., 2020; LeBlanc et al., 2018; Lee et al., 2018; Metskas and Rhoades, 2020; Nasir et al., 2020; Schuler et al., 2016). The dynamic nature of these proteins and their interactions play major roles in numerous cellular processes, including the formation of membrane-less intracellular biomolecular condensates, a new paradigm that presents huge challenges for traditional tools of structural biology (Banani et al., 2017; Choi et al., 2020; van der Lee et al., 2014; Wright and Dyson, 2015). Many IDPs undergo large folding transitions in conjunction with binding to partners, while others remain disordered upon complex formation (Schuler et al., 2020; Wang and Wang, 2019; Wu et al., 2017). SmFRET studies of these systems began more than a decade ago and have tackled increasingly complex systems using more advanced methods, including three-color smFRET or complex labeling schemes (Borgia et al., 2018; Kim and Chung, 2020; Lee et al., 2018; Metskas and Rhoades, 2020; Milles et al., 2015; Nasir et al., 2019; Schuler et al., 2016; Yoo et al., 2020). A recent study that combined smFRET, NMR, and MD simulations to investigate the interaction of H1 with ProTα is highlighted in Figure 9 (Borgia et al., 2018).

Studies of IDPs are even more challenging in heterogeneous environments such as phase-separated mesoscale structures (e.g., membrane-less organelles) or cells (Nasir et al., 2019). Here, the strengths of smFRET would be especially valuable, while the field, at the same time, will benefit from the methodological developments. The ultimate goal is to combine both the structural and dynamic information in order to reduce the ambiguity in the underlying structures of conformational states and to gain detailed information on kinetic pathways between the associated states.

Although we have focused mostly on kinetic studies, smFRET-based structure determination and structural dynamics in this paper, there are a myriad of other new exciting directions where smFRET will have future impact. A detailed description of the various possibilities is beyond the scope of this report, but it is worth mentioning a few of them below.

- Combining FRET with other fluorescence methods: Several groups have combined smFRET with other fluorescence techniques, including protein-induced fluorescence enhancement (PIFE) (Hwang et al., 2011; Hwang and Myong, 2014; Lerner et al., 2016; Ploetz et al., 2016), photoinduced electron transfer (PET) (Haenni et al., 2013), quenchable FRET (Cordes et al., 2010) and stacking-induced fluorescence increase (SIFI) (Morten et al., 2020). The advantages of combining smFRET with other fluorescence-based rulers with higher sensitivity at short distances are obvious – gaining more spatial information on biomolecular systems being measured as well as information on possible synchronized motions between different parts of the biomolecule or biomolecular complex and between different modes of motion. As an example, single-molecule PIFE was used for probing the local structural stabilization in the intrinsically disordered protein α-Synuclein (Chen et al., 2020), which typically appears globally disordered when measured over larger distances using smFRET experiments. Another possibility is combining FRET with information regarding the shape of biomolecules and their assemblies via their translational (Dertinger et al., 2008; Sherman and Haran, 2006) and rotational diffusion (Möckel et al., 2019; Pieper and Enderlein, 2011; Viegas et al., 2020), determined using various FCS modalities. Recently, fluorescence anisotropy- and polarization-resolved FCS were used in integrative studies with non-FRET methods to probe local flexibilities (Möckel et al., 2019) or identify hinge-regions of a protein (Tsytlonok et al., 2020; Tsytlonok et al., 2019).
- Combining FRET with force spectroscopy. Another popular combination is FRET with various manipulation methods including optical tweezers (Hohng et al., 2007), magnetic tweezers (Lee et al., 2010a; Long et al., 2016; Swoboda et al., 2014), tethered particle motion (May et al., 2014), and force spectroscopy by DNA origami (Nickels et al., 2016). The advantages of combining smFRET with force manipulation techniques are obvious – detecting local structural changes or molecular interactions (via smFRET) as well as the global extension of macromolecules (via bead tracking) synchronously under mechanical control.
- Combining FRET with MD simulations. MD simulations have been widely applied with smFRET experiments to provide atomistic insights into the dynamic behavior of biomolecules and their assemblies, as shown in Figure 9 (Barth et al., 2018; Borgia et al., 2018; Holmstrom et al., 2018; Lehmann et al., 2020; Matsunaga and Sugita, 2018; Tsytlonok et al., 2020; Yanez Orozco et al., 2018; Zhao et al., 2010b). The vast information provided by MD simulations often motivates new hypotheses about the functional mechanism that can be tested experimentally via targeted mutations. MD simulations can also be combined with the information provided by smFRET experiments to steer the simulation from one conformational state to the other using accelerated or enhanced sampling techniques (Dimura et al., 2020). To characterize highly dynamic systems, coarse-grained approaches have been applied to intrinsically disordered proteins (Borgia et al., 2018), nucleic acids (Craggs et al., 2019), large chromatin arrays (Kilic et al., 2018) or large DNA origami nanostructures (Bartnik et al., 2020; Khara et al., 2018), to sample the conformational space of the system more efficiently. Alternatively, discrete MD simulations coupled with replica exchange, where discretized potential energies are employed, also assist in accelerating atomistic MD simulations of the ensemble structure of intrinsically disordered proteins (Brodie et al., 2019; Brodie et al., 2017; Chen et al., 2020; Fay et al., 2016; Hadi-Alijanvand et al., 2016; Popov et al., 2019) and holds great promises for being incorporated with single-molecule fluorescence-based technique.
- Combining FRET with imaging. SmFRET can be combined with super-resolution imaging (STED-FRET, Kim et al., 2018b; Tardif et al., 2019; Szalai et al., 2021) or FRET-DNA-PAINT (Deußner-Helfmann et al., 2018; Filius et al., 2020). The combination of fluorescence imaging with spectroscopy makes it possible to detect more species within a pixel of an image, expanding the information that can be extracted from such an experiment. Correlative imaging with electron microscopy, fluorescence and FRET also has the potential to allow the recognition of different subpopulations in the sample, which can then be separated for single-particle reconstructions (Ando et al., 2018; de Boer et al., 2015; Schirra and Zhang, 2014; Verkade and Collinson, 2019).
- SmFRET in live cells. Genetically-encoded fluorescent proteins are the most widely used fluorophores in live-cell imaging. Their maximal brightness and photon yield are, however, limited by excitation-dependent blinking and photobleaching, respectively (Dickson et al., 1997; Seefeldt et al., 2008). Combined with their large size, this makes them non-ideal for quantitative FRET studies. Nevertheless, considerable efforts have been made to extract quantitative FRET information to live-cell imaging data using the fluorescence intensity (Coullomb et al., 2020; Periasamy et al., 2008) or lifetime and anisotropy information (Hinde et al., 2012; Kravets et al., 2016; Kudryavtsev et al., 2007; Liput et al., 2020; Weidtkamp-Peters et al., 2009), and to develop an appropriate dye model for fluorescent proteins (Greife et al., 2016). Recently, the green fluorescent protein has been used for in-cell smFRET measurements in combination with an organic dye attached via the self-labeling protein tag (HaloTag) (Okamoto et al., 2020). To avoid the drawbacks of fluorescent proteins, several groups have shown that smFRET can be performed in live bacterial and eukaryotic cells by using in vitro labeled biomolecules that can be internalized in cells by several means. Electroporation has been shown to work well for the internalization of ssDNA, dsDNA, tRNA and proteins into bacteria and yeast cells (Craggs et al., 2019; Plochowietz et al., 2017; Plochowietz et al., 2016; Plochowietz et al., 2014; Sustarsic et al., 2014; Volkov et al., 2018). Microinjection of labeled molecules is an alternative approach, especially for smFRET in live eukaryotic cells, and has been demonstrated to yield structural information and dynamics from nanoseconds to milliseconds (König et al., 2015; Sakon and Weninger, 2010). With the further development of probes and labeling strategies, and the engineering of better fluorescent proteins (Shaner et al., 2007), there are many exciting possibilities for investigating cellular processes with unprecedented detail (Sustarsic and Kapanidis, 2015).
- SmFRET studies in crowded environments. SmFRET can be used to investigate the influence of the surrounding environment on biomolecules. Such studies can be performed over a drastic range of measurement conditions: from single molecules isolated in solvent cages to molecular environments equivalent to cellular conditions with millimolar concentrations of crowded biomolecules. An important thermodynamic effect of the limited space is minimization of the excluded volume. This (i) influences the hydrodynamic volume of biomolecules with potential consequences for their internal structure, dynamics and functionality and (ii) favors the association state in binding equilibria leading to phase transitions (Banani et al., 2017; Kuznetsova et al., 2014). The profound experimental impact of crowding was confirmed by computer simulations (Nawrocki et al., 2019; Sugita and Feig, 2020) and experimental studies (Gao et al., 2016; Gnutt et al., 2015; Guin and Gruebele, 2019; Möckel et al., 2019; Nasir et al., 2019; Neubauer et al., 2007; Reinkemeier et al., 2019; Zosel et al., 2020b) using labeled molecules as tracers even down to the single-molecule level. In the context of living cells, biomolecular condensates formed by liquid-liquid phase separation have recently been recognized as an important mechanism to spatially organize complex biochemical reactions in membrane-less organelles within the cytoplasm or nucleus (Banani et al., 2017; Hyman et al., 2014). We envision that smFRET studies, especially in combination with integrative experimental approaches, will play a central role in uncovering the dynamic organization and interactions within phase-separated droplets in vitro and in living cells.
- In vitro smFRET of membrane proteins. One class of proteins that remains understudied by structural biology in general is membrane proteins, owing to the complexity of membrane protein production, stabilization and crystallization. As smFRET requires only low amounts of protein to be produced and is performed under experimental conditions that potentially limit solubility issues, it serves a vital role here. Indeed, in recent years, smFRET is increasingly being used to study a variety of membrane proteins, including G-protein-coupled receptors (Gregorio et al., 2017; Olofsson et al., 2014), transporters (Akyuz et al., 2013; Ciftci et al., 2020; Dyla et al., 2017; Fitzgerald et al., 2019; Husada et al., 2018; Terry et al., 2018), and ion channels (Bavi et al., 2016; Wang et al., 2016; Wang et al., 2014). For some recent reviews, see Husada et al., 2015; Martinac, 2017; Quast and Margeat, 2019. However, membrane proteins in a living cell are surrounded by specific lipids, proteins, ion gradients and an electric membrane potential. In addition to investing in intracellular smFRET assays, an important challenge for in vitro smFRET on membrane proteins is to further develop ‘cell-mimicking’ assays.
- SmFRET between multiple chromophores. By measuring the transfer of excitation energy between three or more spectrally different fluorophores, multiple distances are obtained simultaneously, and the correlation of the distances can be determined. Following early ensemble implementations (Haustein et al., 2003; Horsey et al., 2000; Ramirez-Carrozzi and Kerppola, 2001; Watrob et al., 2003; Yim et al., 2012), three- and four-color smFRET experiments have been applied to various static (Clamme and Deniz, 2005; Lee et al., 2007b; Stein et al., 2011) and dynamic systems (Ferguson et al., 2015; Götz et al., in preparation; Hohng et al., 2004; Lee et al., 2010c; Lee et al., 2010b; Morse et al., 2020; Munro et al., 2010; Ratzke et al., 2014; Vušurović et al., 2017; Wasserman et al., 2016). FRET to many acceptors has also been reported (Krainer et al., 2015; Uphoff et al., 2010). Multi-color FRET experiments, however, remain challenging, in particular for diffusion-based experiments, because of the increased shot-noise, and the more complex FRET efficiency calculations and corrections. Recent advances in this respect include the development of a photon distribution analysis for three-color FRET to extract three-dimensional distance distributions (Barth et al., 2019) and a maximum likelihood approach applied to the study of fast protein folding (Kim and Chung, 2020; Yoo et al., 2020; Yoo et al., 2018). Further progress in multiple-chromophore smFRET will require expanding the useable spectral range to the near infra-red (which requires better fluorophores and detectors in that region) and measurement of the single-molecule spectra (Lacoste et al., 2000; Squires and Moerner, 2017) rather than the use of individual channels.
- SmFRET with nanomaterials. Emerging structurally synthesized and targeted specific nanomaterials such as quantum dots (QDs) (Jamieson et al., 2007), aggregation-induced emission (AIE) nanoparticles (Hong et al., 2011), and nitrogen-vacancy centers in diamond (Schirhagl et al., 2014; Tisler et al., 2011) have made it possible to implement chemically engineered fluorophores for a wide range of applications in structural biology investigations and, more specifically, in FRET-related studies (Börsch et al., 2009; Medintz et al., 2003; Oh et al., 2005; Shi et al., 2006; Soleimaninejad et al., 2017).
- SmFRET with plasmonics. Placing fluorescent dyes close to metallic nanostructures in ‘plasmonic hotspots’ increases the detectable signal of a single molecule into the megahertz region (Acuna et al., 2012; Grabenhorst et al., 2020). Recent work has shown the possibility of plasmon-assisted FRET (Baibakov et al., 2020; Baibakov et al., 2019; Bohlen et al., 2019). Excitingly, it has recently been shown that tryptophan fluorescence of proteins can be detected with single-molecule resolution in zero-mode waveguides (Barulin et al., 2019), paving the way toward studies using intrinsic labels.

## Epilogue

In this article, we have summarized current perspectives on the status of the smFRET field, limitations that still need to be overcome, and joint efforts towards the adoption of consistent methodologies and open-science practices. While this article encourages a discussion regarding optimal smFRET practices, it is important to remember that, as scientists, we should value independence of thought and creativity. Hence, our recommendations should be taken as constructive suggestions, and it is important to realize that many biological questions can be answered using multiple approaches. On the one hand, the reproducibility and reliability of smFRET measurements are currently limited by the variety of approaches taken to calculate the FRET efficiency and the resulting inter-dye distance. Combining years of experience from various experts in an open discussion can help us, as a community, to improve the methodology and overcome many of its challenges. On the other hand, it is important to be open to new ideas and approaches. Here is where open scientific practices can help the community to quickly exchange data and analysis approaches to test new ideas. Such a community effort is necessary to consolidate the role of smFRET as a useful tool in various fields and to jointly move the field forward. Our hope is that these efforts will benefit not only the smFRET community, but also the structural biology community and science in general.

## Appendix 1

### Simulation software

- simFCS (https://www.lfd.uci.edu/globals/) was developed for simulating various modalities in fluorescence, beginning with FCS. Using two channels, the simulation of burst analysis smFRET data can be performed. The concentration, diffusion coefficient and molecular brightness in different channels and dynamics between them can be adjusted.
- PyBroMo (https://github.com/tritemio/PyBroMo) is a Python-based software package for simulating free-diffusion single-molecule fluorescence detection, including differences in translational diffusion coefficients, in concentrations, or in dye brightnesses and exchange dynamics between conformational states (currently between two states). This approach has been employed in a few recent works (Ingargiola et al., 2017; Lerner et al., 2018b; Hagai and Lerner, 2019).
- PAM software package (https://www.cup.uni-muenchen.de/pc/lamb/software/pam.html). The PAM software package is a MATLAB-based software that can simulate smFRET experiments of freely diffusing or immobilized molecules, including fluorescence lifetime and anisotropy as well as exchange dynamics between multiple conformational states (up to 8) Szalai et al., 2021 Schrimpf et al., 2018).
- The Burbulator software within the MFD program package, programed in LabVIEW (Dimura et al., 2016; Felekyan et al., 2012; Kalinin et al., 2010b) (https://www.mpc.hhu.de/software/3-software-package-for-mfd-fcs-and-mfis), can simulate smFRET experiments (with and without diffusion) combined fluorescence lifetime and anisotropy as well as exchange dynamics between multiple conformational states (up to 8). These tools have been applied to benchmark novel quantitative analysis methods to obtain structural and kinetic information.
- Fretica (https://schuler.bioc.uzh.ch/programs/) enables the simulation of single-molecule multichannel-detection of immobilized molecules and mixtures of freely diffusing species, including a dynamic exchange between an arbitrary number of conformational states (König et al., 2015; Zosel et al., 2018).
- The MATLAB -based MASH-FRET software package (Börner et al., 2018) (https://rna-fretools.github.io/MASH-FRET/) has been applied to evaluate transition detection and state identification algorithms used in particular for time-binned smFRET trajectories (Hadzic et al., 2018) as well as for spot detection in single-molecule videos (SMV).
- The python-based software DeepFRET (Thomsen et al., 2020) comes with a trace simulator capable of simulating traces with 17 adjustable features that include the number of FRET states, their values, noise level, transition probability and more (https://github.com/hatzakislab/DeepFRET-GUI).
